# Integrative Transcriptomics and Machine Learning Nominate *IL15*- and *PPP2R1A*-Centered Programs in PTSD Using Pathway-Informed Autonomic–Cardiac Gene Prioritization

**DOI:** 10.3390/ijms27198710

**Published:** 2026-09-29

**Authors:** Yihan Guo, Dongdong Shi, Lanying Liu, Zhen Wang

**Affiliations:** 1 School of Integrative Medicine, Shanghai University of Traditional Chinese Medicine, Shanghai 201203, China; yohoo97@126.com (Y.G.); sdd_921@sjtu.edu.cn (D.S.); balindaliu@163.com (L.L.); 2 Shanghai Key Laboratory of Psychotic Disorders, Shanghai Mental Health Center, Shanghai Jiao Tong University School of Medicine, Shanghai 201108, China; 3 Shanghai Mental Health Center, Shanghai Jiao Tong University School of Medicine, Shanghai 200030, China

**Keywords:** post-traumatic stress disorder, autonomic dysregulation, transcriptomics, machine learning, single-nucleus RNA sequencing, IL15, PPP2R1A

## Abstract

Post-traumatic stress disorder (PTSD) is associated with immune and autonomic disturbances, but molecular programs linking PTSD-related blood transcriptional signals with autonomic–cardiac biology remain incompletely characterized. The public Gene Expression Omnibus (GEO) cohorts analyzed here did not directly measure palpitations or autonomic dysfunction. Peripheral-blood transcriptomes from GSE81761 and GSE63878 were integrated with a prespecified pathway-derived autonomic–cardiac gene set constructed from Kyoto Encyclopedia of Genes and Genomes (KEGG) pathways. Genes overlapping the curated pathway set were assessed using functional enrichment and protein–protein interaction (PPI) analyses. Multi-algorithm comparison and a separate random-forest/SHapley Additive exPlanations (SHAP) analysis were performed for internal model assessment and biological prioritization. GSE64813 and GSE97356 were analyzed using targeted single-gene analyses and cohort-specific multivariable logistic models. Model performance was examined using repeated nested cross-validation, a fully nested sensitivity analysis, and locked-transfer testing to GSE64813 and GSE97356. *Interleukin 15 (IL15)* and *protein phosphatase 2 scaffold subunit Aalpha (PPP2R1A)* were evaluated using external-cohort analyses, immune-cell deconvolution, and descriptive postmortem hippocampal single-nucleus data. Among 1679 nominally significant PTSD-associated candidate genes, 92 overlapped the curated pathway set and were enriched for cytokine signaling, chemotaxis, apoptosis, calcium transport, and PP2A-related functions. PPI analysis yielded 37 recurrent candidate hub genes. The original model comparison ranked support-vector machine (SVM) the highest across the merged and source-cohort summaries [mean area under the receiver-operating-characteristic curve (AUC) 0.853], but these estimates represent internal discovery-stage performance. *IL15* was recurrently prioritized by network-based analyses, whereas *PPP2R1A* was a component of an enriched phosphatase-related module. *IL15* and *PPP2R1A* showed modest single-gene effects in GSE64813 (AUC 0.566 and 0.612) and GSE97356 (AUC 0.558 and 0.593). Cohort-specific refitted models showed apparent AUC values of 0.834 and 0.749, respectively. Repeated nested cross-validation conditional on the preselected 29-gene feature set identified L2-regularized logistic regression as the best-performing algorithm (mean AUC = 0.690). Importantly, a more stringent fully nested sensitivity analysis, in which differential-expression screening and pathway intersection were repeated within each outer training fold, yielded a mean repeated outer-cross-validated AUC of 0.593 [standard deviation (SD) = 0.033; range, 0.541–0.625], indicating limited predictive performance. Locked-transfer AUCs were 0.664 in GSE64813 and 0.534 in GSE97356. Single-nucleus summaries suggested donor- and nucleus-type-dependent expression patterns for *IL15* and *PPP2R1A*. These findings identify *IL15*-related cytokine signaling and *PPP2R1A*-related phosphatase regulation as candidate molecular programs linking PTSD-associated transcriptional variation with pathway-derived autonomic–cardiac biology. The results should therefore be interpreted as hypothesis-generating rather than direct molecular evidence for unmeasured palpitation symptoms. Prospective validation in independent, clinically well-characterized cohorts with standardized autonomic and cardiac phenotyping is warranted.

## 1. Introduction

Post-traumatic stress disorder (PTSD) is a psychiatric condition characterized by intrusive memories, avoidance, negative alterations in cognition and mood, and increased arousal following exposure to traumatic events [1,2]. Although PTSD is primarily considered a condition of the central nervous system, its effects extend beyond the brain. Many individuals with PTSD report systemic manifestations, including palpitations, exaggerated cardiovascular responses, dyspnea, and excessive sweating, features that may reflect altered autonomic regulation. These clinical manifestations are linked to dysregulated sympathetic and parasympathetic activity, impaired neuroendocrine stress signaling, and peripheral immune activation; however, the underlying molecular mechanisms in these interconnected processes remain poorly explored [3,4,5,6,7,8].

Transcriptomic analyses of peripheral blood have revealed dysregulation in inflammatory pathways, interferon-related signaling, and stress-response networks in patients with PTSD [9,10,11,12]. Despite these promising observations, the reported molecular signals remain heterogeneous across independent cohorts, suggesting a complex underlying biology that is unlikely to be captured by a single biomarker [9,10]. Therefore, analytical approaches that combine pathway-informed prioritization with systematic evaluation across multiple datasets may provide a more credible strategy for identifying more reproducible molecular signatures associated with PTSD [10,12]. Furthermore, observations derived from blood should also be interpreted within the broader neurobiological context, as several genes perform distinct functions depending on the cellular environment, such as immune cells, neurons, glial cells, and vascular compartments. Hence, combining peripheral transcriptomic data with observations derived from relevant brain and neurovascular cell populations may provide broader insights into the underlying biological mechanisms in PTSD [13,14].

Palpitations are commonly reported in PTSD, but their underlying mechanisms are likely to be heterogeneous. These symptoms may reflect cardiac arrhythmia, autonomic hyperarousal, altered interoceptive processing, or combinations of these processes. Notably, the publicly available GEO cohorts analyzed in this study were not selected on the basis of documented palpitations, and palpitations were not systematically assessed as a clinical phenotype. We therefore did not attempt to infer a molecular signature of palpitations. Rather, we used a prespecified, pathway-derived autonomic–cardiac gene set to examine whether PTSD-associated blood transcriptional signals overlap with pathways involved in autonomic and cardiovascular regulation, immune signaling, and neuroendocrine stress responses. This approach supports biological hypothesis generation, rather than evidence that the identified genes are directly associated with an unmeasured cardiac symptom. This interpretation is also consistent with previous genetic and multi-omic investigations reporting shared biological pathways between PTSD susceptibility and hematological or cardiometabolic traits [15,16,17,18,19].

Accordingly, we integrated peripheral blood transcriptomic data with PPI network analysis, machine-learning strategies, postmortem hippocampal single-nucleus transcriptomic data, immune-cell deconvolution, and functional-network analyses. The aim was to prioritize candidate molecular programs at the interface of PTSD-related blood transcription alterations and autonomic–cardiac pathway biology, thereby providing a foundation for future mechanistic studies and validation in cohorts with detailed clinical and autonomic–cardiovascular phenotyping [20].

## 2. Results

### 2.1. A PTSD-Associated Blood Signature Intersects Autonomic–Cardiac Pathways

**Figure 1:**
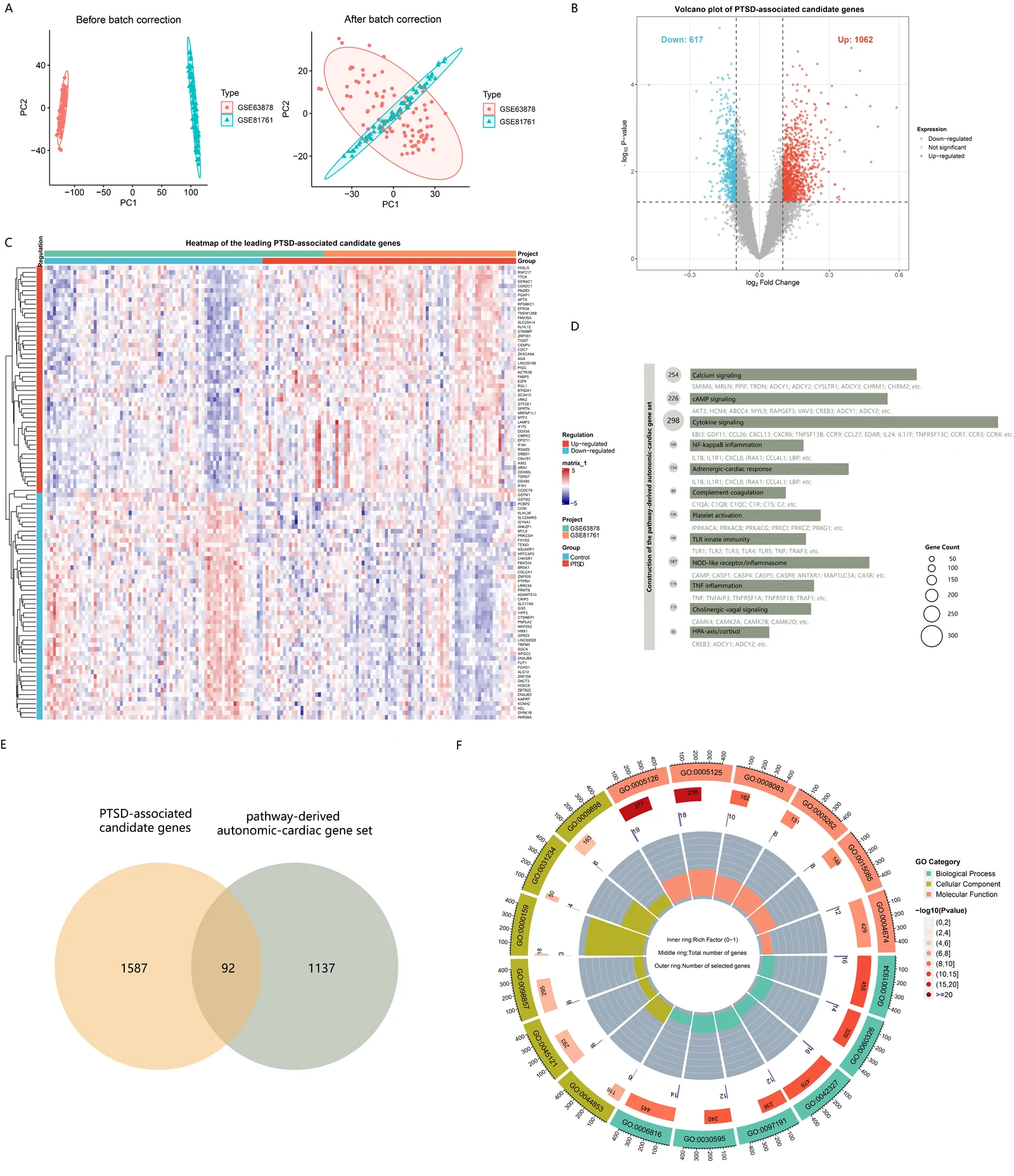
Functional characterization of PTSD-associated genes and their overlap with the pathway-derived autonomic–cardiac gene set. (**A**) PCA before and after batch-effect correction. (**B**) Volcano plot of PTSD-associated candidate genes. (**C**) Heatmap of the leading PTSD-associated candidate genes. (**D**) Composition of the pathway-derived autonomic–cardiac gene set. (**E**) Overlap between PTSD-associated candidate genes and pathway-derived autonomic–cardiac gene sets. (**F**) GO enrichment of the 92 overlapping genes. From the inner to the outer ring, the circular plot represents the Rich Factor (0–1), the number of selected genes, and the total number of genes, respectively. The color of the outer ring represents −log10^(P value)^, with darker red indicating higher −log10^(P value)^ values. The colors of the GO term labels indicate the three GO categories: Biological Process, Cellular Component, and Molecular Function.

PCA showed significant separation between the two discovery datasets before batch-effect correction. After ComBat correction, the samples demonstrated substantially greater overlap, indicating attenuation of dataset-related variation while retaining biological heterogeneity (Figure 1A). Using the prespecified exploratory threshold of nominal *p* < 0.05 and |log2 fold change| > 0.1, 1679 genes were identified as nominally significant PTSD-associated candidate genes, including 1062 upregulated and 617 downregulated genes (Figure 1B). These genes were not interpreted as a definitive FDR-controlled DEG set; rather, they were retained as an exploratory candidate list for pathway-informed prioritization. The heatmap showed consistent expression patterns across the two discovery datasets (Figure 1C), with prominent changes involving genes related to cellular stress, immune sensing, mitochondrial regulation, and ion-channel activity.

The pathway-derived autonomic–cardiac gene set was specified before inspection of differential-expression results and comprised 1229 unique genes from KEGG pathways involved in adrenergic signaling, cAMP and calcium signaling, cholinergic signaling, cortisol regulation, inflammatory responses, coagulation, platelet, and other processes relevant to autonomic–cardiac biology (Figure 1D). Among the 1679 nominally significant PTSD-associated candidate genes, 92 overlapped with this prespecified gene set, corresponding to 5.48% of the nominal candidate genes and 7.49% of the pathway-derived autonomic–cardiac gene set (Figure 1E). The overlapping genes included *MYL9*, *AIM2*, *MAP2K2*, *FASLG*, *STIM2*, *IL15*, *ROCK2*, *KCNQ1*, *PIK3R3*, *TLR3*, *TBK1*, *P2RX1*, *ORAI1*, *PANX1*, *PPP2R1A*, *PRKCA*, *MAP3K7*, *JAK2*, *CASP1*, *CXCL8*, *CXCL10*, and *IFNG* (Table S4). Collectively, these genes link diverse biological processes relevant to the pathway framework, including inflammatory signaling, calcium homeostasis, intracellular phosphorylation, and cardiac electrophysiological regulation.

GO enrichment analysis revealed 590 substantially enriched GO terms after FDR correction, including 520 BP, 43 MF, and 27 CC terms (Figure 1F). The most enriched biological processes included positive regulation of protein phosphorylation, chemotaxis, extrinsic apoptotic signaling, calcium-ion transport, and NF-κB signaling. Prominent molecular function terms were cytokine receptor binding and cytokine activity, whereas the leading enriched cellular-component terms included membrane rafts and membrane microdomains. The PP2A complex, represented by *PPP2R5A*, *PPP2R1A*, and *PPP2R2B*, showed a 36.16-fold enrichment (RichFactor = 0.1667; GeneRatio = 3/92; adjusted *p* value = 0.003), indicating that phosphatase-dependent signaling is a prominent component of the shared gene set.

### 2.2. PPI Network Identifies a Distributed Immune-Signaling Core

**Figure 2:**
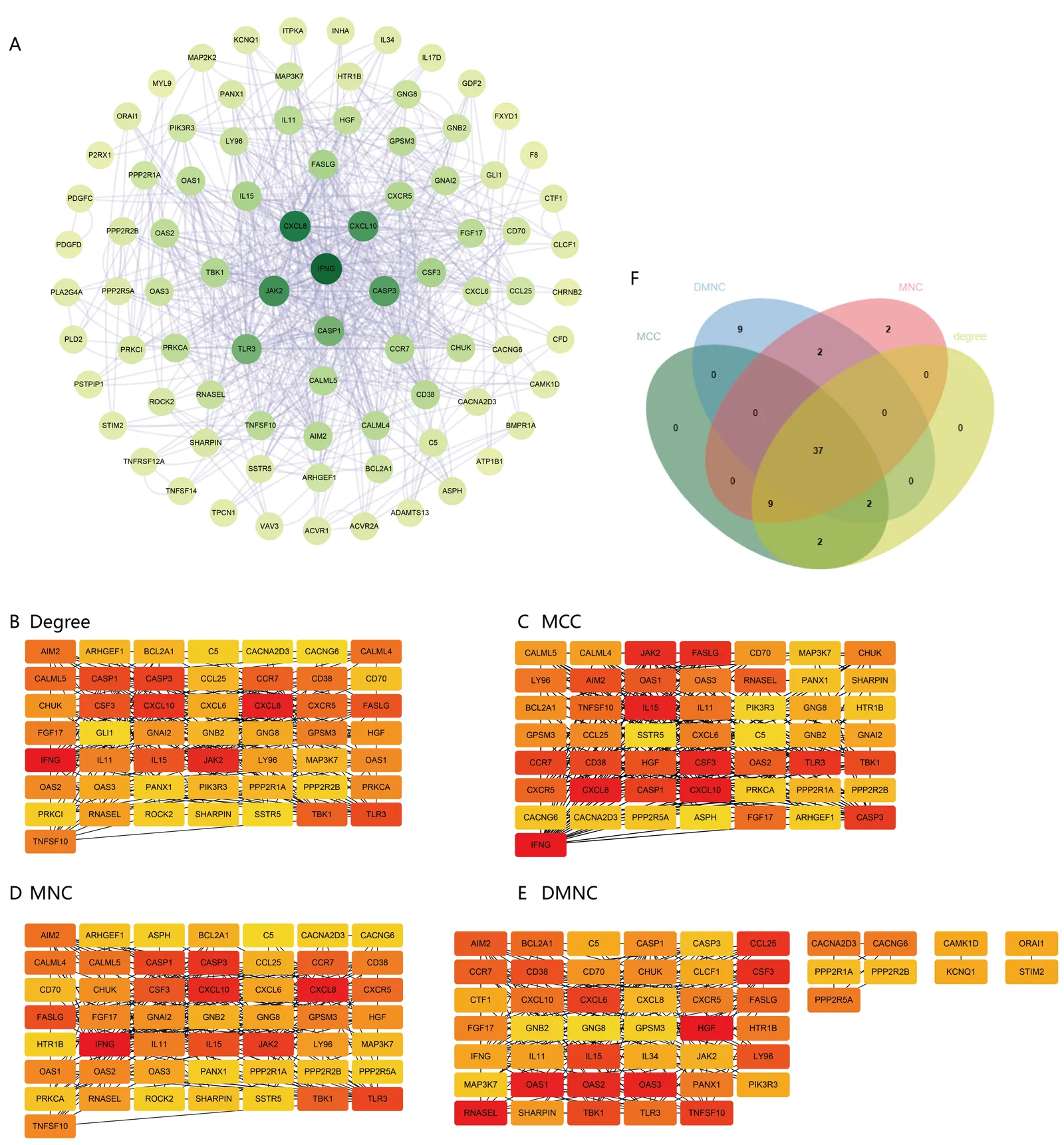
PPI topology and robust hub-gene identification. (**A**) PPI network of the 92 shared genes. (**B**–**E**) Top 50 nodes ranked by degree, MCC, MNC, and DMNC. (**F**) Intersection of the four rankings, yielding 37 recurrent hub genes.

The PPI network established from the 92 genes shared between the nominally significant PTSD-associated candidate genes and the pathway-derived autonomic–cardiac gene set showed a densely connected central region surrounded by less-connected peripheral nodes (Figure 2A). The network consisted of 92 nodes and 338 edges, with an average node degree of 7.35 and an average local clustering coefficient of 0.583. PPI enrichment was statistically significant (*p* < 1 × 10^−16^), indicating greater connectivity among these proteins than expected for a random protein set of comparable size. The densely connected core was enriched in cytokines, chemokines, innate immune sensors, and cell-death regulators, whereas proteins involved in calcium signaling, ion transport, and growth-factor pathways were distributed in the network periphery. Ranking genes using degree, MCC, MNC, and DMNC algorithms identified 37 candidate hub genes that were consistently ranked among the top 50 by all four ranking approaches (Figure 2B–F).

Functional annotation of these hub genes showed four major biological categories: (1) cytokine and chemokine signaling, including *IFNG*, *CXCL8*, *CXCL10*, *CXCL6*, *CSF3*, *IL15*, *IL11*, *CCL25*, *CCR7*, *CXCR5*, and *CD70*; (2) innate immune and interferon signaling, including *TLR3*, *TBK1*, *LY96*, *OAS1*, *OAS2*, *OAS3*, and *RNASEL*; (3) inflammasome activation and cell-death pathways, including *AIM2*, *CASP1*, *CASP3*, *FASLG*, *TNFSF10*, *PANX1*, and *BCL2A1*; and (4) intracellular signaling and phosphatase regulation, including *JAK2*, *MAP3K7*, *CHUK*, *SHARPIN*, *PPP2R1A*, and *PPP2R2B*.

Out of these genes, *IL15* was consistently ranked highly across the four centrality measures, placing fourth by MCC, eighth by degree and MNC, and tenth by DMNC. *IL15* was directly connected to *IFNG*, *JAK2*, *CXCL10*, and *FASLG* in the PPI network. *PPP2R1A* showed interaction with the phosphatase-regulatory component of the network, indicating its role in intracellular signaling pathways represented in the shared gene set.

### 2.3. Machine-Learning Results and Leakage-Aware Sensitivity Analysis

**Figure 3:**
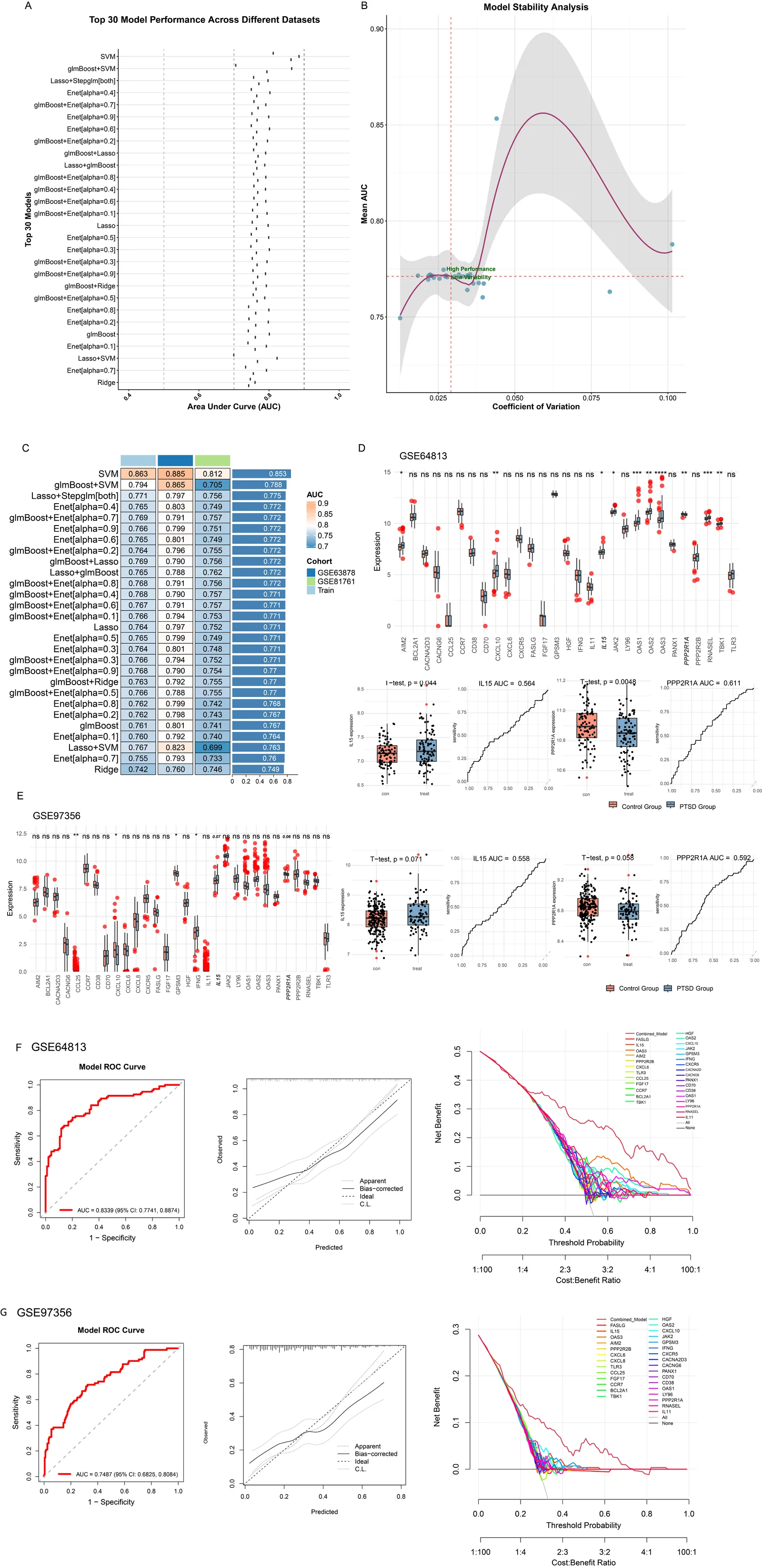
Original machine-learning and external-cohort analyses. (**A**) AUCs of leading model combinations in the discovery framework. (**B**) Algorithm-performance stability across the merged discovery matrix and its source cohorts. Each point represents the mean AUC and coefficient of variation for an individual model across datasets. The magenta fitted curve shows the overall relationship between model performance and variability, and the gray shaded area represents the confidence interval around the fitted curve. The red dashed horizontal and vertical lines indicate the predefined reference thresholds for mean AUC and coefficient of variation, respectively. The annotated region indicates models with relatively high performance and low variability. (**C**) Heatmap of model AUCs. (**D**,**E**) Candidate-gene expression and single-gene ROC analyses in GSE64813 and GSE97356.Boxplots show the distribution of gene expression levels, and red dots indicate outliers identified by the boxplot. Statistical significance between groups was assessed using a t-test, with ns indicating no significance and *, **, *** and **** indicating increasing levels of statistical significance. (**F**,**G**) ROC, bootstrap calibration, and exploratory DCA for multivariable logistic models fitted separately in GSE64813 (29 available genes) and GSE97356 (30 genes). Because panels F and G use predictions from the same samples used for coefficient estimation, their AUCs and decision curves represent apparent cohort-specific performance rather than locked external validation. AUC, area under the receiver-operating-characteristic curve; DCA, decision-curve analysis.

In the original discovery framework, the SVM showed the most consistent discrimination, with AUC values of 0.863 in the merged discovery dataset, 0.885 for GSE63878, and 0.812 for GSE81761 (mean AUC of 0.853; Figure 3A–C and Table S3). Because both source cohorts contributed to model development, and because outcome-informed candidate-gene selection was completed before the original resampling-based algorithm comparison, these performance estimates should be interpreted as internal discovery-stage measures rather than independent validation. In the locked-model sensitivity analysis, the model achieved a mean outer-fold AUC of 0.690 (0.4603 to 0.9206) (SD = 0.117) across 50 outer test folds (Table S5). The range denotes the minimum and maximum AUC values observed across the 50 outer test folds and is not a 95% confidence interval. The relatively wide dispersion of outer-fold performance indicates variability in model discrimination across resampled test sets. The fully nested sensitivity analysis yielded a mean repeated outer-cross-validated AUC of 0.593 (SD = 0.033; range, 0.541–0.625), providing a more conservative estimate than the previous nested evaluation conditional on the preselected 29-gene feature set (AUC = 0.690). *IL15* and *PPP2R1A* were retained in 25/25 and 24/25 outer folds, respectively (Table S11).

An independent random forest model incorporating the 37 candidate hub genes was established to facilitate SHAP-based feature interpretation. Repeated cross-validation identified an optimal mtry value of 2, yielding a cross-validated AUC of 0.679 (Table 1 and Table S6). This model was used only for biological interpretation and was established independently of both the original SVM ranking and the locked 29-gene sensitivity.

SHAP analysis of the RF model identified *FASLG* and *IL15* as the most influential features, with mean absolute SHAP values of 0.0221 and 0.0218, respectively. Their bootstrap 95% confidence intervals largely overlapped (*FASLG*, 0.0194–0.0249; *IL15*, 0.0195–0.0238), showing no clear difference in their relative importance. The other top-ranked genes included *OAS3*, *AIM2*, *PPP2R2B*, *CXCL6*, *CXCL8*, *TLR3*, *CCL25*, and *FGF17*, completing the top 10 highest-ranked features. These top 10 genes accounted for 36.1% of SHAP importance, whereas approximately 28 genes were required to reach 80% of cumulative importance. These results indicate that the model obtained its predictive signal from the combined contribution of multiple genes rather than a single-gene classifier (Table S7).

### 2.4. External Cohorts Reproduce Modest Single-Gene Effects but Refitted Multigene Estimates Are Optimistic

Because GSE64813 contained repeated measurements that were analyzed as individual expression profiles, the *p* values and bootstrap confidence intervals reported for analyses involving this cohort do not account for within-participant correlation and should therefore be interpreted as exploratory profile-level estimates. In the GSE64813 cohort, *IL15* expression was higher in PTSD-labeled profiles than in the controls (*p* = 0.044), although its discriminatory power was modest (AUC = 0.566). In contrast, *PPP2R1A* expression was lower in PTSD-labeled samples (*p* = 0.0048) and achieved slightly greater discriminatory performance (AUC = 0.612; Figure 3D, Table S8). The same bidirectional pattern was found in GSE97356. However, neither targeted comparison reached the conventional threshold for statistical significance (*IL15* *p* = 0.071, AUC = 0.558; *PPP2R1A* *p* = 0.058, AUC = 0.593; Figure 3E, Table S8). Overall, the effect sizes were modest, suggesting that neither gene alone provides adequate discriminatory performance to serve as a standalone biomarker.

The cohort-specific 29-gene model fitted to GSE64813 yielded an apparent AUC of 0.834 (bootstrap 95% CI 0.774–0.887), whereas the 30-gene model fitted to GSE97356 achieved an apparent AUC of 0.749 (95% CI 0.682–0.808) (Figure 3F,G, Table S9). Model calibration and DCA were conducted within the same cohorts used for model construction. Although the candidate gene set captured cohort-specific patterns, these observations do not reflect model transportability. In the locked sensitivity analysis, AUC values were 0.664 (95% CI 0.586–0.744) in GSE64813 and 0.534 (95% CI 0.460–0.605) in GSE97356 (Table S10), indicating modest predictive performance when examined under a more stringent validation framework.

### 2.5. Postmortem Hippocampal Single-Nucleus Data Place IL15 and PPP2R1A in Distinct Cellular Contexts

**Figure 4:**
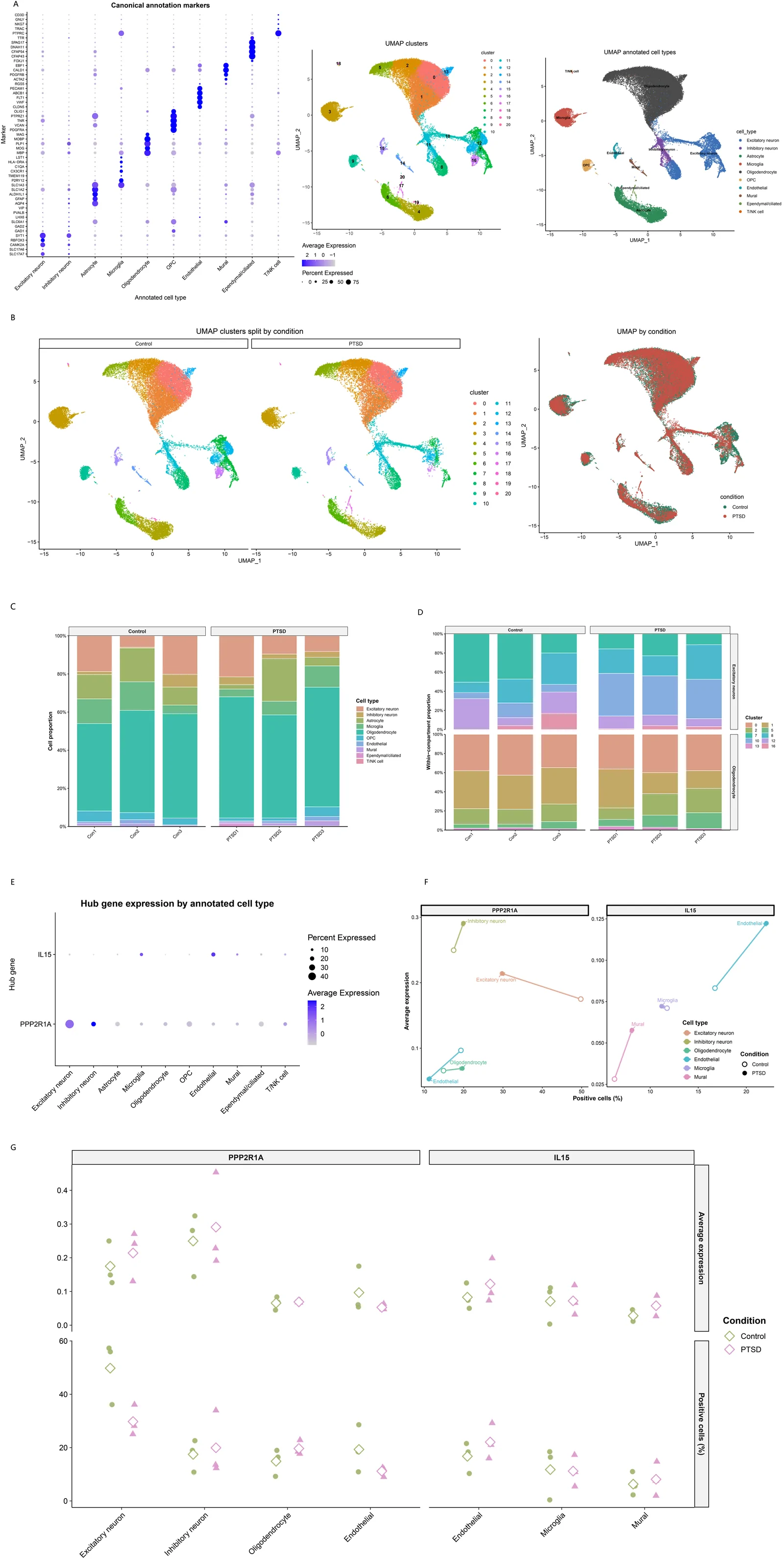
Postmortem hippocampal single-nucleus landscape and cell-type-specific IL15 and PPP2R1A expression levels. (**A**) UMAP of 48,065 nuclei colored by cluster and annotated cell type. (**B**) UMAP distributions by condition. (**C**) Donor-level nucleus-type proportions. (**D**) Within-lineage cluster composition for excitatory neurons and oligodendrocytes. (**E**) Dot plots of IL15 and PPP2R1A expression across cell types. (**F**) Mean expression and positive-nucleus fractions in representative populations. (**G**) Donor-level summaries in selected cell types. Filled circles represent individual Control donors, filled triangles represent individual PTSD donors, and open diamonds indicate the corresponding group means. All condition comparisons are descriptive.

The processed GSE313257 dataset contained 48,065 nuclei obtained from three PTSD and three control donors. The 21 clusters were annotated into 10 major cell types: excitatory neurons, inhibitory neurons, oligodendrocytes, astrocytes, microglia, OPCs, endothelial cells, mural cells, ependymal/ciliated cells and T/NK cells (Figure 4A). Oligodendrocyte nuclei represented the largest cell population (55.57%), followed by excitatory-neuronal (14.60%), astrocytic (11.45%), and microglial (8.79%).

UMAP visualization demonstrated substantial overlap between PTSD and control nuclei, with no clear group separation (Figure 4B). At the donor level, PTSD samples showed a descriptively higher proportion of oligodendrocytes (mean, 60.10% versus 51.41%) and a slight increase in endothelial and mural cells, whereas astrocytic, microglial, and excitatory-neuronal cells and OPCs were less abundant (Figure 4C). However, considerable variation was found among individual donors. Within individual cell lineages, excitatory neuron cluster 10 and oligodendrocyte clusters 2, 5, and 13 were more abundant in PTSD samples, whereas excitatory neuron clusters 7, 12, and 16 and oligodendrocyte cluster 1 were less prominent (Figure 4D). The descriptive differences observed suggest possible variation in cell composition but cannot demonstrate group-level differences because of the small number of biological donors.

*PPP2R1A* expression was most prominent in neuronal cell populations, with the highest mean expression found in inhibitory neurons and the largest proportion of expressing cells in excitatory neurons (Figure 4E,F). Overall expression was similar between conditions, but cell-type-specific summaries suggested higher mean expression and a lower positive fraction of excitatory neurons in PTSD, together with a higher proportion of expressing oligodendrocytes and a lower proportion of expressing endothelial cells (Figure 4G). These observations are compatible with cell-type-specific heterogeneity rather than a uniform change across all cell populations.

*IL15* showed low overall expression but was relatively enriched in endothelial, microglial, and mural cells (Figure 4E,F). PTSD samples showed descriptively higher *IL15* expression in endothelial cells, both in terms of mean normalized expression (0.1230 versus 0.0718) and the proportion of expressing cells (22.68% versus 14.34%). A similar pattern was found in mural cells (0.0547 versus 0.0319). Microglial *IL15* expression was descriptively lower in PTSD (Figure 4G). Given the small number of donors (n = 6), these results should be considered descriptive and suggest donor- and cell-type-specific variability rather than evidence of population-level or causal differences.

### 2.6. IL15 and PPP2R1A Map to Complementary Immune and Signaling Programs

**Figure 5:**
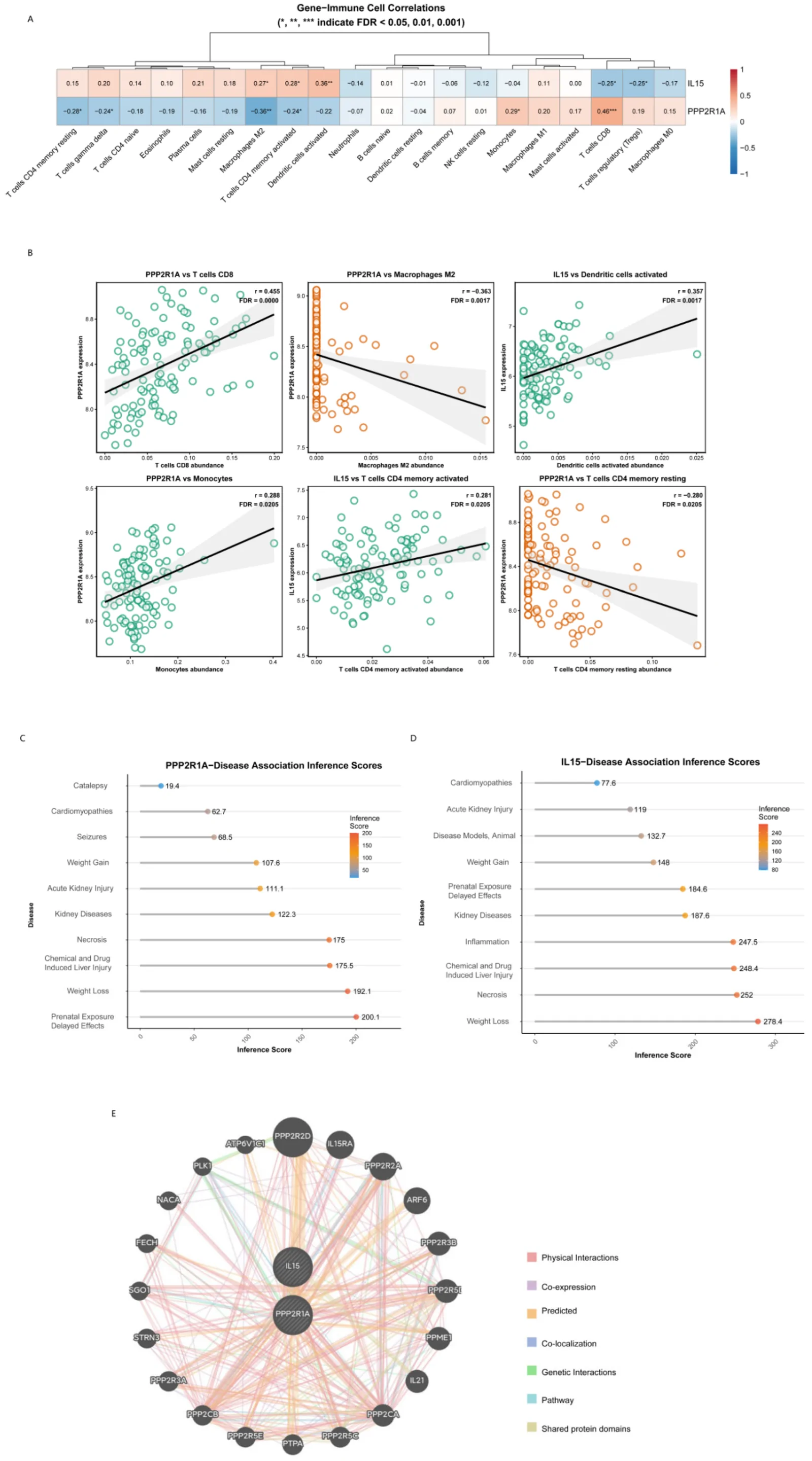
Immune and functional context of *IL15* and *PPP2R1A*. (**A**) Spearman’s correlations between candidate-gene expression and CIBERSORT-estimated immune-cell proportions. (**B**) Representative gene–cell associations.Green circles indicate positive correlations, whereas orange circles indicate negative correlations. Black lines represent the fitted linear relationships, with shaded areas showing the corresponding confidence intervals. (**C**,**D**) Leading CTD gene–disease associations for *PPP2R1A* and *IL15*. (**E**) GeneMANIA network generated with *PPP2R1A* and *IL15* as seed genes. Asterisks in (**A**) denote FDR <0.05, <0.01 and <0.001.

CIBERSORT analysis of the batch-corrected discovery cohort (GSE81761 and GSE63878) identified variation in estimated peripheral immune-cell composition across expression profiles. *PPP2R1A* expression was positively associated with the estimated abundance of CD8+ T-cells (Spearman’s r = 0.455, FDR < 0.001) and monocytes (r = 0.288, FDR = 0.0205), while showing negative correlations with M2 macrophages (r = −0.363, FDR = 0.0017) and resting CD4+ memory T cells (r = −0.280, FDR = 0.0205). In contrast, *IL15* expression was positively associated with the estimated proportion of activated dendritic cells (r = 0.357, FDR = 0.0017) and activated CD4+ memory T cells (r = 0.281, FDR = 0.0205). These correlations indicated distinct associations between *IL15* or *PPP2R1A* expression and estimated peripheral immune cell composition. However, they were not adjusted for all potential confounders, including PTSD status, dataset source, sampling time, batch effects, or participant-level repeated measurements, and therefore should be interpreted as descriptive associations rather than causal or directional relationships (Figure 5A,B). Both candidate-gene expression and estimated immune-cell proportions were sourced from the same bulk transcriptomic matrix; therefore, these correlations are not independent measurements and may partly reflect cell-composition-associated expression structure.

CTD analysis showed overlapping annotation profiles for *IL15* and *PPP2R1A*, including kidney injury, cardiomyopathy, chemically induced tissue injury, and necrosis (Figure 5C,D). *IL15* was additionally associated with inflammatory phenotypes, whereas *PPP2R1A* demonstrated a strong association with neurological annotations, such as seizures. Because CTD ranking scores are influenced by the quantity and distribution of available evidence, these results were used to provide biological context rather than to infer candidate gene causality.

GeneMANIA network analysis revealed that physical interactions account for the highest proportion of the integrated network evidence (77.64% of the total network weight; Figure 5E). *PPP2R1A* was embedded within a phosphatase-related module containing regulatory and catalytic PP2A components, including *PPP2R2A/D*, *PPP2R3A/B*, *PPP2R5C/D/E*, *PPP2CA/B*, *PPME1*, and *PTPA*. In contrast, *IL15* was primarily connected to cytokine-related genes, including *IL15RA* and *IL21*. Together, these analyses underscore the involvement of phosphatase-regulatory and cytokine-related functional modules associated with the two candidate genes.

## 3. Discussion

This study integrated pathway-informed transcriptomic analysis, PPI network analysis, machine-learning approaches, external-cohort assessment, and postmortem hippocampal single-nucleus RNA sequencing to explore biological processes shared between PTSD-related blood transcriptional alterations and pathways associated with autonomic–cardiac regulation. These integrated analyses identified candidate genes involved in cytokine signaling, innate-immune responses, calcium transport, cell-death pathways, and phosphatase-mediated signaling. *IL15* and *PPP2R1A* emerged as candidate genes representing cytokine-related and PP2A-associated regulatory programs, respectively. Importantly, these findings indicate exploratory pathway overlap and network prioritization, not direct molecular evidence for palpitation or autonomic dysfunction as unmeasured clinical symptoms.

The 92 genes shared between the nominally significant PTSD-associated candidate set and the pathway-derived autonomic–cardiac gene set underscore biological processes that may facilitate interaction between systemic stress responses and autonomic–cardiac regulation. Functional enrichment further revealed pathways associated with cytokine activity, chemotaxis, NF-κB signaling, calcium transport, ion-channel regulation, and membrane microdomains. These findings indicate convergence of immune activation and vascular signaling relevant to cardiovascular physiology. Nevertheless, the GEO cohorts did not include standardized clinical measures of palpitation or autonomic dysfunction; therefore, the results should be interpreted as evidence of overlap with a curated pathway-based framework rather than a molecular signature of clinically adjudicated palpitations.

The machine-learning findings underscore an important distinction between biological candidate prioritization and the ability of a predictive model to generalize across cohorts. The original SVM model showed good discrimination within the combined discovery dataset and its component cohorts. Likewise, AUC values of 0.834 and 0.749 were observed for GSE64813 and GSE97356 using logistic regression models developed and assessed separately within each cohort. As these models were trained and tested using the data from the same cohort, the observed performance indicates apparent, cohort-specific discrimination rather than independent external validation. In contrast, the more stringent validation analyses yielded substantially lower performance estimates. The fully nested sensitivity analysis, in which differential-expression screening and pathway intersection were repeated within each outer training fold, yielded a mean repeated outer-cross-validated AUC of 0.593 (SD = 0.033; range, 0.541–0.625), indicating limited predictive performance under this validation framework. Locked-transfer evaluation yielded AUCs of 0.664 in GSE64813 and 0.534 in GSE97356. These findings underscore that the current transcriptomic model has not yet demonstrated robust cross-cohort predictive generalizability. The reduction in performance across locked external cohorts may reflect multiple sources of heterogeneity, including transcriptomic platform, preprocessing, cellular composition, trauma context, clinical definition, medication, and comorbidity profiles. The present data cannot separate the individual contribution of these factors. These findings support current recommendations from the TRIPOD+AI and PROBAST+AI guidelines, which highlight rigorous control of information leakage, independent external evaluation, and transparent reporting of prediction models [21,22]. Although the cohort-specific models indicated dataset-specific expression patterns, they should not be considered evidence of a clinically validated diagnostic model or broad cross-cohort generalizability.

*IL15* and *PPP2R1A* were observed as biologically relevant candidate genes rather than validated standalone biomarkers for PTSD. *IL15* was ranked among the most influential features in SHAP analysis and occupied a central position within the cytokine interaction network. Its association with estimated immune cell populations and its descriptive expression pattern in neurovascular cell types are compatible with the established role of *IL-15* in regulating T/NK-cell homeostasis and immune signaling [23,24]. *PPP2R1A* was identified within an enriched PP2A-related signaling module and demonstrated lower expression in PTSD-labeled samples in both external cohorts. Although PP2A plays a significant role in calcium homeostasis, β-adrenergic signaling, and synaptic function, these observations reinforce further investigation of *PPP2R1A* in the context of PTSD [25,26]. Nevertheless, the discriminatory performance of the individual genes remained limited (AUC = 0.5–0.6), suggesting that neither gene demonstrated sufficient discriminatory potential to support interpretation as a standalone diagnostic biomarker.

The single-nucleus analysis provided anatomical context while highlighting the importance of donor-level inference. Although descriptive differences were found in oligodendrocyte and excitatory neuron subclusters between PTSD and control donors, and *IL15* expression was relatively enriched in neurovascular cell populations, these findings should be interpreted with caution. Previous single-cell and single-nucleus studies have supported substantial cell-type specificity in PTSD-associated brain alterations, particularly involving astrocytes, hippocampal immune responses, and glial cell populations [27,28]. However, the present findings are based on only three donors per group and used a preprocessed object without re-estimation of raw-data quality control metrics; spatial transcriptomic analyses were also unavailable. As a result, these results offer preliminary evidence for donor- and cell-type-specific expression patterns rather than definitive evidence of altered cellular composition or spatial localization. Pseudobulk or mixed-effects analyses in larger donor cohorts are needed to minimize pseudoreplication and improve the robustness of cell-type-specific inferences [29,30].

The permissive threshold (nominal *p* < 0.05 and |log2 fold change| > 0.1) used in the discovery analysis requires careful interpretation. The 1679 genes identified were intended to provide an exploratory candidate pool for subsequent pathway-informed prioritization and should not be interpreted as a transcriptome-wide FDR-controlled differential-expression signature. Hence, the prioritization of *IL15* and *PPP2R1A* indicates convergence across pathway membership, network or feature-importance analyses, and external-cohort assessment rather than FDR-significant differential expression alone. These findings support further investigation of these genes and associated molecular programs but do not establish them as validated PTSD biomarkers.

This study integrates multiple publicly available databases and combines transcriptomic, PPI network, ML, immune-deconvolution, and single-nucleus analyses while differentiating biological interpretation from prediction modeling. However, several limitations should be considered before clinical translation. First, the GEO cohorts did not include standardized measurements of palpitations, heart-rate variability, autonomic function, or related arrhythmia outcomes; thus, the findings cannot be interpreted as direct molecular evidence for palpitations or autonomic dysfunction in PTSD. Second, cohorts differed in transcriptomic platform, trauma exposure, medication use, comorbidities, demographic features, and preprocessing, all of which may have contributed to biological and technical heterogeneity. Third, participant-level identifiers were not consistently retained in the harmonized matrices used for all analyses, so within-participant dependence and possible participant-level fold leakage could not be fully excluded. Fourth, the 1679-gene discovery list was based on a permissive nominal threshold and should be treated as an exploratory candidate set rather than a definitive FDR-controlled DEG signature. Fifth, the locked-model sensitivity analysis reduced model-refitting bias but remained conditional on candidate-gene selection performed before resampling and therefore does not represent a fully feature-selection-nested validation. Finally, CIBERSORT-derived immune proportions are indirect computational estimates and should be interpreted cautiously. The observed correlations between *IL15* or *PPP2R1A* expression and estimated immune-cell proportions were derived from the same bulk transcriptomic data and were not adjusted for PTSD status, dataset source, sampling time, batch effects, or repeated measurements. Consequently, these associations may show differences in cellular composition or other cohort-level sources of heterogeneity rather than direct gene-specific effects on immune-cell abundance. Validation using independent cohorts with measured cell counts or single-cell data may help establish the cellular context of these candidate genes.

Future investigations should assess these observations in larger, well-characterized cohorts with longitudinal follow-up. Recent longitudinal blood transcriptomic studies and a cytokine meta-analysis reinforce the importance of capturing dynamic immune responses and clinical heterogeneity throughout the course of PTSD [31,32]. Cardiovascular evidence and early autonomic-intervention studies further support incorporating standardized cardiac outcomes and physiological measurements rather than inferring autonomic involvement solely from pathway-based analyses [33,34]. Larger single-nucleus cohorts analyzed using pseudobulk or donor-level mixed-effect models could further assess the cell-type-specific expression patterns reported for *IL15* and *PPP2R1A* [35]. Integrating transcriptomic data with proteomic and metabolomic cohorts may help distinguish core PTSD signals from multisystem comorbidity [36]. Finally, future prospective prediction studies should include predefined clinical covariates, fully locked preprocessing pipelines, and a comprehensive assessment of calibration and transportability.

## 4. Material and Methods

### 4.1. Study Design and Cohort Allocation

Pathways involved in autonomic and cardiac regulation, as well as immune and neuroendocrine stress responses, were selected from KEGG to construct the pathway-derived autonomic–cardiac gene set [37]. The GSE81761 and GSE63878 datasets were combined because both included comparable PTSD and control expression profiles. Pooling these datasets increased the sample size and statistical power for detecting PTSD-associated transcriptional alterations while reducing the influence of dataset-specific variation. The combined discovery cohort comprised 87 PTSD-labeled and 75 control-labeled expression profiles. After data harmonization, normalization, and batch-effect correction, PTSD-associated candidate genes were identified and intersected with the pathway-derived autonomic-cardiac gene set. The resulting genes were subsequently analyzed using Gene Ontology (GO) enrichment analysis, protein–protein interaction (PPI) network analysis, and machine-learning approaches. Two independent datasets, GSE64813 and GSE97356, were used for external-cohort assessment and locked-transfer evaluation.

**Figure 6:**
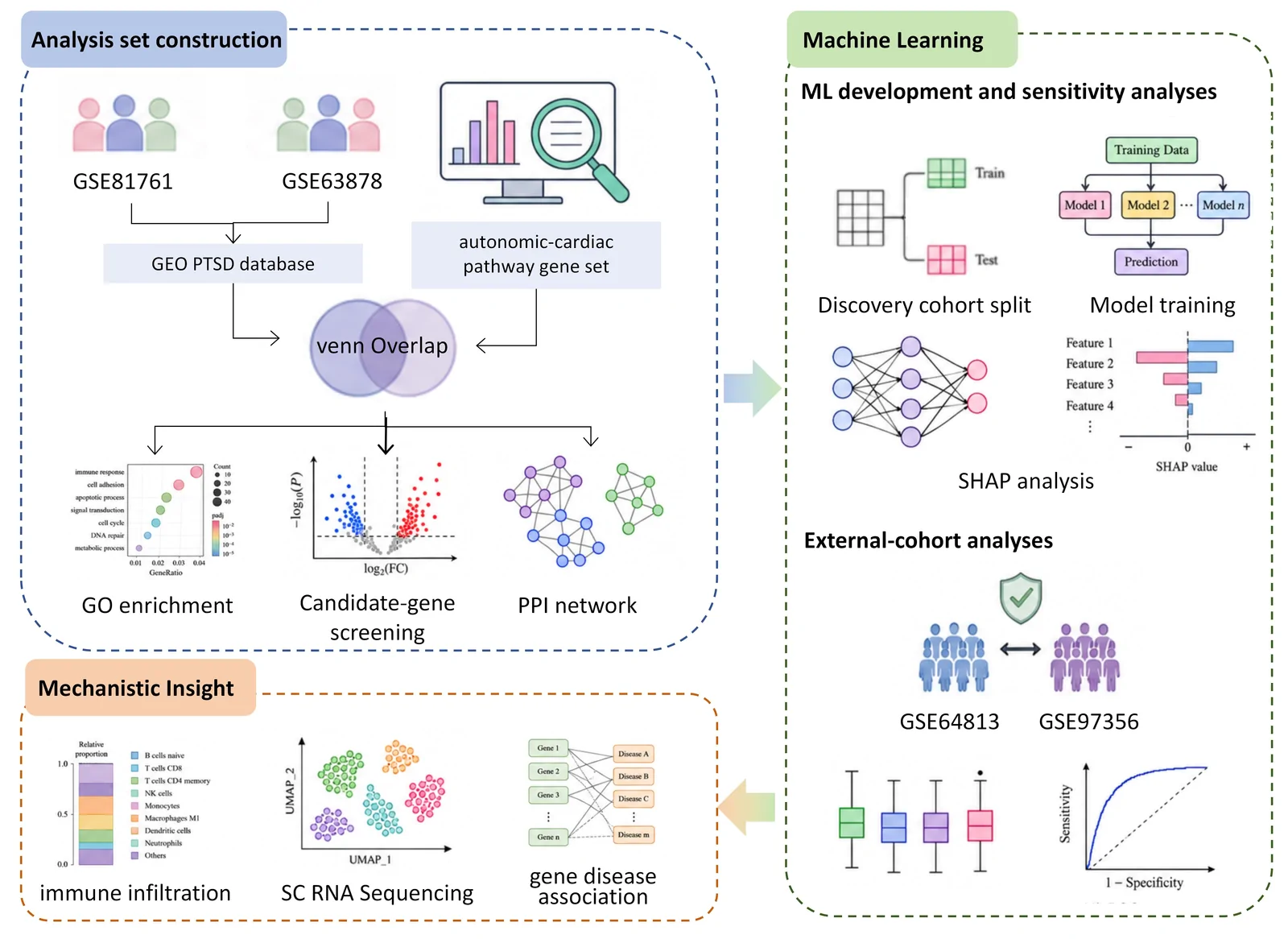
Study design and analytical workflow. Peripheral-blood transcriptomic datasets (GSE81761 and GSE63878) were combined to form the discovery cohort and analyzed in conjunction with the pathway-derived autonomic–cardiac gene set. Hub gene screening incorporated differential-expression analysis, GO enrichment, PPI network analysis, and machine-learning strategies. The original discovery framework identified SVM as the highest-performing algorithm, whereas a separate random-forest/SHAP analysis was applied for biological feature prioritization. Candidate genes were further examined in GSE64813 and GSE97356 using targeted single-gene analyses, cohort-specific multivariable logistic regression, and locked-transfer sensitivity analysis. *IL15* and *PPP2R1A* were selected for further characterization, and their biological context was assessed using postmortem hippocampal single-nucleus RNA sequencing (GSE313257), immune-cell deconvolution, GeneMANIA network analysis, and the CTD. CTD, Comparative Toxicogenomics Database; CV, cross-validation; PPI, protein–protein interaction; RF, random forest; SHAP, SHapley Additive exPlanations; SVM, support vector machine.

*IL15* and *PPP2R1A* were selected for subsequent analysis because they represent key components of cytokine signaling and PP2A-dependent phosphorylation regulation, respectively. Their cell-type-specific expression was examined in GSE313257, and their additional molecular associations were assessed through immune-cell deconvolution, GeneMANIA network analysis, and the Comparative Toxicogenomics Database (CTD) (Figure 6).

### 4.2. Bulk Transcriptomic Datasets and Preprocessing

PTSD-related transcriptomic datasets were retrieved from the GEO database [38]. The GEO search was last audited on 6 September 2026 using the terms “post-traumatic stress disorder” OR “posttraumatic stress disorder” OR PTSD. Search results were restricted to Homo sapiens and GEO Series entries. Inclusion criteria were (1) a minimum of 10 samples per analytical group; (2) availability of PTSD/case-labeled and non-PTSD/control-labeled groups; (3) human expression data from whole blood, peripheral-blood leukocytes, or closely related peripheral-blood fractions; (4) the primary subjects of the study are adults. Exclusion criteria included non-human studies, methylation-only or miRNA-only assays, intervention-only designs without a non-PTSD comparison group, non-blood tissue for bulk transcriptomics, insufficient group size, absence of usable binary PTSD labels, or duplicate SuperSeries/sample-level entries. The search found 48 records. Following the predefined eligibility criteria, four bulk peripheral-blood transcriptomic datasets were retained for analysis. The included and excluded records, with reasons for exclusion, are provided in Supplementary Table S1, and the screening process is summarized in Supplementary Figure S1.

GSE81761 and GSE63878 were used for the discovery analysis, whereas GSE64813 and GSE97356 were used as external-cohort datasets. GSE313257 was not included in the bulk transcriptomic workflow and was used only for descriptive postmortem single-nucleus analysis. The main characteristics of the bulk transcriptomic cohorts are available in Table 2. Additional cohort clinical information, such as age, sex, type of trauma, PTSD diagnostic criteria, timing of blood sampling, treatment status, psychotropic medication use, and major comorbidities, is summarized in Table S2 where available. Throughout the study, sample counts refer to expression-matrix sample profiles and should not be interpreted as counts of unique participants. For GSE63878 and GSE64813, repeated measurements were retained in their original form and examined as individual expression profiles rather than being collapsed to the participant level.

Normalized expression matrices from GSE81761 and GSE63878 were restricted to genes shared between the two datasets, and duplicate gene entries were consolidated by averaging their expression values. To reduce dataset-specific technical variation, batch effects were corrected using ComBat implemented in the sva package (v3.60.0) [39,40], with dataset source specified as the batch variable and PTSD status included as a biological covariate in the design matrix. This design allowed ComBat to adjust for dataset-associated technical variation while preserving expression differences associated with PTSD status. Principal-component analysis (PCA) was performed before and after batch correction to assess the reduction of dataset-related variation and the preservation of biological group structure.

Differential-expression analysis between PTSD and control profiles was performed using the batch-corrected discovery dataset and the limma package ((v3.68.0) [41]. For exploratory pathway-informed candidate prioritization, genes with a nominal *p* < 0.05 and |log2 fold change| > 0.1 were retained [42]. This deliberately permissive threshold was used to increase sensitivity for the downstream pathway-informed discovery analysis rather than to define a transcriptome-wide significant differential-expression signature. Genes meeting these criteria were therefore described as nominally significant PTSD-associated candidate genes rather than definitive differentially expressed genes (DEGs). Overall expression patterns were visualized using a volcano plot. For heatmap visualization, genes were ranked according to log2 fold change, and the genes with the largest positive and negative fold changes were selected and row-wise standardized using Z scores.

### 4.3. Construction of the Pathway-Derived Autonomic–Cardiac Gene Set

To construct the pathway-derived autonomic–cardiac gene set, KEGG pathways were prespecified a priori, before inspection of the PTSD differential-expression results. Pathway selection was based on established biological relevance to four domains: autonomic neurotransmission, cardiac signaling and electrophysiology, neuroendocrine stress regulation, and immune-inflammatory mechanisms with cardiovascular relevance [43]. This prespecified strategy reduced the potential for post hoc selection of pathways based on the observed PTSD-associated transcriptional signals.

The selected pathways included adrenergic signaling in cardiomyocytes (hsa04261), cAMP signaling pathway (hsa04024), calcium signaling pathway (hsa04020), cholinergic synapse (hsa04725), cortisol synthesis and secretion (hsa04927), cytokine-cytokine receptor interaction (hsa04060), TNF signaling pathway (hsa04668), NF-κB signaling pathway (hsa04064), Toll-like receptor signaling pathway (hsa04620), NOD-like receptor signaling pathway (hsa04621), complement and coagulation cascades (hsa04610), and platelet activation (hsa04611).

Gene annotations for the selected pathways were retrieved from KEGG using the KEGGREST package (v1.52.0) in July 2026. Gene identifiers were standardized to official gene symbols and Entrez Gene identifiers, and duplicate genes occurring in more than one pathway were removed to generate a unique gene set. The final pathway-derived autonomic–cardiac gene set comprised 1229 unique genes. Official KEGG pathway names and identifiers, pathway-specific gene counts, retrieval date, and the complete nonredundant or unique gene list are provided in Supplementary2_KEGG.xlsx. PTSD-associated nominal candidate genes were then intersected with the pathway-derived autonomic–cardiac gene set to identify candidate genes potentially linking PTSD-related blood transcriptional alterations with autonomic–cardiac pathway biology.

### 4.4. Gene Ontology Enrichment Analysis

After eliminating duplicate entries, gene symbols were converted to Entrez Gene identifiers using the org.Hs.eg.db annotation package (v3.23.1), and the unmapped genes were excluded from subsequent analyses. GO over-representation analysis was performed using the enrichGO function in the clusterProfiler package (v4.20.0) to assess enrichment across the biological process (BP), cellular component (CC), and molecular function (MF) ontologies [44,45]. GO terms were considered significantly enriched at both a nominal *p* < 0.05 and a Benjamini–Hochberg false discovery rate (FDR)-adjusted *p* < 0.05 [42]. Significant terms were prioritized according to adjusted *p* value, GeneRatio, and gene count, and representative enriched categories were visualized using circular plots.

### 4.5. PPI Network and Hub-Gene Analysis

The genes shared between PTSD-associated nominal candidate genes and the pathway-derived autonomic–cardiac gene set were mapped to STRING (v11; Homo sapiens) to form a PPI network using a combined interaction score of ≥200. A relatively permissive combined-score threshold was used to retain broader network coverage and potentially informative interactions for exploratory topology-based analysis. To reduce reliance on any single network-ranking criterion, candidate hubs were required to rank among the top 50 nodes across all four cytoHubba algorithms. The resulting interaction network was analyzed in Cytoscape (v3.10.2) [46]. To detect hub genes, nodes were ranked using four algorithms implemented in the cytoHubba plugin: degree, maximal clique centrality (MCC), maximum neighborhood component (MNC), and density of maximum neighborhood component (DMNC) [47]. Genes appearing in the top 50 ranked nodes across all four approaches were designated as recurrent candidate hub genes and were included in the downstream analyses.

### 4.6. Machine-Learning Development and Sensitivity Analysis

The discovery datasets were assessed for log transformation, normalized where appropriate, restricted to genes shared across datasets, and corrected for batch effects. Gene features were then standardized before model development. A two-stage ML framework was implemented. In the first stage, after standardization and Gaussian noise augmentation (mean = 0, SD = 0.01; seed = 123), the prespecified algorithms were applied in variable-selection mode to the discovery training data to generate algorithm-specific candidate feature sets; a feature set containing all input genes was also retained. This outcome-informed candidate-feature selection was performed before the resampling-based model comparison. In the second stage, each classifier was refitted using its corresponding selected feature set. Models retaining fewer than three genes after fitting were excluded from subsequent assessment. The evaluated algorithms included elastic net, Least Absolute Shrinkage and Selection Operator (LASSO), ridge regression, stepwise logistic regression, support vector machine (SVM), linear discriminant analysis (LDA), glmBoost, partial least-squares generalized linear modeling (PLS-GLM), random forest, gradient boosting, XGBoost and naive Bayes [48,49,50,51].

Regularization parameters were optimized using 10-fold cross-validation. The random forest model was trained using 1000 trees with a minimum node size of 5, whereas boosting algorithms were applied using the hyperparameter setting used in the original analysis workflow. Model discrimination was assessed using the area under the receiver operating characteristic curve (AUC) [52]. Algorithm performance was evaluated using the merged discovery dataset and two source cohorts (GSE81761 and GSE63878). The resulting performance measures were considered estimates of internal consistency rather than independent validation (Table S3).

In the original workflow, the files labelled train.csv and test.csv contained the same underlying discovery profiles after sample-prefix normalization, indicating that an independent test set was not available. To address this, a separate locked-model sensitivity analysis was performed using repeated nested cross-validation, consisting of a 10-fold outer cross-validation repeated five times and a 5-fold inner cross-validation for hyperparameter optimization. To improve compatibility across discovery and external datasets, the features were limited to the 29 candidate genes shared among all cohorts. For each candidate algorithm, hyperparameter optimization was performed within the inner cross-validation loop, and predictive performance was subsequently evaluated on the corresponding outer test folds. Algorithm performance was compared using mean outer-fold AUC, after which the best-performing algorithm and its optimized hyperparameters were locked and evaluated in the external cohorts without outcome-guided model refitting.

To assess potential optimism associated with feature selection before resampling, we additionally performed a reduced fully nested sensitivity analysis focused on the SVM classifier, in which differential-expression screening and pathway intersection were repeated within each outer training fold, followed by hyperparameter tuning in inner cross-validation. Full details are provided in the Supplementary Methods.

### 4.7. SHAP Analysis

To facilitate biological interpretation of the full set of 37 candidate genes identified through PPI network analysis, a separate auxiliary random forest (RF) model was developed specifically for SHAP-based interpretation. This auxiliary model was independent of the RF model described in Section 4.6, which was trained with 1000 trees for predictive modeling. For the SHAP-specific auxiliary RF model, hyperparameter tuning was performed using repeated 10-fold cross-validation with five repetitions. Candidate mtry values of 2, 4, 6, 8, and 10 were evaluated, and the value yielding the highest cross-validated AUC was selected. The final auxiliary RF model was then trained with 500 trees and used exclusively for SHAP analysis of the 37 candidate genes. This model was distinct from the RF model described in Section 4.6, the original SVM classifier, and the locked 29-gene sensitivity model.

SHAP values were computed using the permutation-based kernelshap algorithm and visualized with the shapviz package (v0.10.4) [53]. Feature importance was determined as the mean absolute SHAP value for each gene. Uncertainty in the importance measures was evaluated using 100 bootstrap resamples to generate 95% percentile confidence intervals. Genes were then ranked based on their mean absolute SHAP values, and cumulative-importance thresholds of 80% and 90% were calculated to determine the relative contribution of the highest-ranking features.

*IL15* and *PPP2R1A* were selected for focused downstream characterization based on complementary discovery-stage evidence: *IL15* showed consistently high PPI-network centrality and ranked among the leading features in the RF/SHAP analysis, whereas *PPP2R1A* represented the enriched PP2A-related phosphatase-regulatory module. Thus, the two genes were carried forward as biologically complementary candidates representing cytokine signaling and phosphorylation control, rather than as the two highest-ranked predictive features.

### 4.8. External-Cohort Analyses

The external evaluation cohorts included GSE64813 (94 PTSD-labeled and 94 control-labeled expression profiles) and GSE97356 (81 PTSD-labeled and 201 control-labeled expression profiles). Differences in candidate-gene expression between PTSD and control groups were assessed using two-sided *t* tests. ROC curves and bootstrap confidence intervals were calculated using the pROC package (v1.19.1). For GSE64813, repeated measurements were retained and analyzed as individual expression profiles rather than being collapsed to the participant level. Because within-participant dependence was therefore not modeled, the *p* values from the two-sided tests and the bootstrap confidence intervals derived from analyses involving this cohort should be interpreted as exploratory profile-level estimates rather than participant-level inferential statistics. To characterize cohort-specific multigene signal structure, separate multivariable logistic regression models were refitted de novo within each cohort using the rms::lrm function and the candidate genes available in that dataset. Because model coefficients were estimated and performance was assessed in the same cohort, these models were not considered external validation of a prespecified prediction model. Their discrimination, calibration, and decision-curve results were therefore interpreted as apparent cohort-specific performance. Nomograms were generated for descriptive purposes, and bootstrap resampling was used to assess within-cohort calibration (1000 resamples) and decision-curve behavior (100 resamples across threshold probabilities from 0 to 1) [54,55]. Evaluation of model transportability was conducted within the locked-model sensitivity analysis using the locked model.

### 4.9. Single-Nucleus Data and Preprocessing

A processed Seurat object from the postmortem hippocampal single-nucleus RNA-sequencing dataset GSE313257 was analyzed using the Seurat framework (Seurat package v5.5.1) [56]. The dataset contained 48,065 nuclei and 31,585 RNA features obtained from three control and three PTSD donors (Table 3). Gene expression analyses were conducted using the RNA assay. The integrated data representation and dimensionality reduction results, such as PCA, t-SNE, and UMAP, were retained for downstream analyses. The dataset also included 21 cell clusters, numbered 0–20, which were used for cell-type annotation and expression analyses.

### 4.10. Nucleus-Type Annotation

Cell clusters were manually annotated using a canonical marker-based approach. The expression profiles of established marker genes were examined for each of the 21 pre-existing clusters and compared with established markers of central-nervous-system and immune cell populations. Based on concordant expression of these canonical markers, clusters were assigned to excitatory neurons, inhibitory neurons, astrocytes, microglia, oligodendrocytes, oligodendrocyte progenitor cells (OPCs), endothelial cells, mural cells, ependymal/ciliated cells, and T/natural-killer (T/NK) cells. No automated cell-type annotation algorithm was used. The resulting cluster-level annotations were propagated to individual nuclei and used for subsequent nucleus-type-specific analyses (Table 4).

### 4.11. Nucleus-Type-Resolved Expression of IL15 and PPP2R1A

The expression levels of *IL15* and *PPP2R1A* were confirmed in the RNA assay, and normalized expression values were retrieved using the FetchData function. Expression of *IL15* and *PPP2R1A* was summarized based on disease status, annotated cell type, donor, and cell cluster. Summary measures included nucleus count, mean and median normalized expression levels, and the proportion of nuclei with detectable expression (normalized expression greater than zero).

Because only three donors were available in each group, the donor rather than the individual nucleus was treated as the biological unit of replication. To avoid pseudoreplication, individual nuclei were not considered independent biological replicates, and no nucleus-level statistical assessment was interpreted as evidence of differential expression between PTSD and control donors. Condition-related expression patterns were therefore summarized at the donor level and interpreted descriptively.

### 4.12. Immune-Cell Deconvolution

CIBERSORT was applied to the batch-corrected expression matrix from the discovery cohort (GSE81761 and GSE63878) to estimate relative immune-cell proportions. Deconvolution was performed using the LM22 leukocyte signature matrix with 1000 permutations and quantile normalization enabled [57]. Gene-expression values for the selected candidate genes were matched to the estimated immune-cell proportions by sample identifier. Immune-cell populations showing no variation across samples were excluded. Samples were excluded if more than 10% of values were missing in either of the two matched data components: candidate-gene expression values or estimated immune-cell proportions. No samples were excluded solely on the basis of CIBERSORT deconvolution *p* values or goodness-of-fit metrics; therefore, all resulting cell-fraction estimates were retained for exploratory correlation analyses.

Spearman rank correlations were used to assess associations between *IL15* and *PPP2R1A* expression and the estimated immune-cell proportions. Correlation analyses were performed only for variable pairs with at least three valid observations and non-zero variance. *p* values were adjusted using the Benjamini-Hochberg procedure, and an FDR-adjusted *p* value < 0.05 was considered statistically significant. Because harmonized participant identifiers, exact blood-sampling time, medication exposure, and comorbidity covariates were not consistently available across the discovery matrices, partial correlation or mixed-effects models adjusting for all potential confounders could not be implemented reliably. Correlation matrices were hierarchically clustered using the Ward.D2 algorithm, and the six strongest associations, ranked by the absolute Spearman correlation coefficient, were visualized with fitted trend lines; these plots were used for descriptive visualization only and not for causal inference.

### 4.13. GeneMANIA and CTD Analyses

Functional interaction networks for *IL15* and *PPP2R1A* were established using GeneMANIA (version 3.5.3, accessed July 2026), with query genes specified as Homo sapiens genes [58]. The resulting network integrated multiple sources, such as co-expression, physical interaction, genetic interaction, co-localization, shared pathway, and protein-domain similarity. The relative contribution of each evidence category was summarized using the network weights generated by GeneMANIA.

Gene–disease associations for *IL15* and *PPP2R1A* were retrieved from CTD (accessed July 2026) and ranked based on the CTD inference score [59]. The 10 highest-ranked associations for each gene were visualized. As CTD scores are affected by the volume and structure of the available annotations, these analyses were used to provide biological context rather than causal inference.

## 5. Conclusions

These integrative analyses prioritized *IL15*-associated cytokine signaling and *PPP2R1A*-related phosphatase regulation as candidate molecular programs shared between PTSD-associated blood transcriptional signals and pathway-derived autonomic–cardiac biology. The fully nested sensitivity analysis yielded an AUC of 0.593, indicating limited predictive performance, while locked-transfer evaluation further demonstrated attenuation across external cohorts. These findings highlight the need for prospective validation in independent, clinically well-characterized cohorts with standardized autonomic and cardiac phenotyping before diagnostic utility can be considered.

## Figures and Tables

**Table 1:** Cross-validation results for the auxiliary random-forest model.

mtry	ROC	Sensitivity	Specificity	ROC SD	Sensitivity SD	Specificity SD
2	0.6785	0.6367	0.5354	0.1073	0.1684	0.1620
4	0.6671	0.6478	0.5543	0.1152	0.1613	0.1737
6	0.6620	0.6228	0.5721	0.1240	0.1715	0.1542
8	0.6651	0.6203	0.5675	0.1243	0.1693	0.1676
10	0.6614	0.6253	0.5557	0.1237	0.1792	0.1479

**Table 2:** Main characteristics of the bulk transcriptomic cohorts.

Characteristic	GSE81761	GSE63878	GSE64813	GSE97356
Platform	GPL570	GPL6244	GPL11154	GPL11154
Organism	Homo sapiens	Homo sapiens	Homo sapiens	Homo sapiens
Sample source	Whole blood	Peripheral-blood leukocytes	Peripheral-blood leukocytes	Whole blood
PTSD/case-labeled samples	n = 39	n = 48	n = 94	n = 81
Control-labeled samples	n = 27	n = 48	n = 94	n = 201
Data type	Microarray	Microarray	RNA sequencing	RNA sequencing
Analytical role	Discovery	Discovery	External-cohort assessment	External-cohort assessment

**Table 3:** Single-nucleus donor composition.

Group	Sample	Nuclei
Control	Con1	7610
Control	Con2	7796
Control	Con3	10,927
PTSD	PTSD1	7698
PTSD	PTSD2	7356
PTSD	PTSD3	6678

**Table 4:** Cluster-to-nucleus-type annotation.

Cell Type	Clusters	Nuclei	Representative Markers
Excitatory neuron	7, 8, 10, 12, 16	7017	RBFOX3, CAMK2A, SYT1, DLGAP1
Inhibitory neuron	11	1583	GAD1, GAD2, SLC6A1
Astrocyte	4, 6, 19	5504	SLC1A2, AQP4, GFAP, GPC5
Microglia	3	4227	P2RY12, TMEM119, C1QA, HLA-DRA, PTPRC
Oligodendrocyte	0, 1, 2, 5, 13	26,708	MBP, MOG, PLP1, MOBP
OPC	9	1660	PDGFRA, VCAN, TNR, PTPRZ1
Endothelial cell	15	570	CLDN5, VWF, FLT1, ABCB1
Mural cell	14	598	RGS5, ACTA2, EBF1, CALD1
Ependymal/ciliated cell	17, 20	136	FOXJ1, CFAP43, CFAP44, CFAP54, DNAH11, SPAG17, TTR
T/NK cell	18	62	PTPRC, TRAC, NKG7, GNLY

## Data Availability

The bulk-transcriptomic and single-nucleus datasets analyzed in this study are publicly available in the Gene Expression Omnibus under accession numbers GSE81761, GSE63878, GSE64813, GSE97356, and GSE313257. The analysis code and derived outputs for the original multi-algorithm model comparison and SVM ranking, RF–SHAP interpretation, and external-cohort analyses, together with the feature lists, sample metadata, predictions, and performance tables, are publicly available in Zenodo at https://doi.org/10.5281/zenodo.21621096.

## Supplementary Materials

The following supporting information can be downloaded at https://www.mdpi.com/article/10.3390/ijms27198710/s1.

## Abbreviations

The following abbreviations are used in this manuscript:

AUC	area under the receiver-operating-characteristic curve
CI	confidence interval
CTD	Comparative Toxicogenomics Database
DCA	decision-curve analysis
DEG	differentially expressed gene
FDR	false discovery rate
GEO	Gene Expression Omnibus
GO	Gene Ontology
KEGG	Kyoto Encyclopedia of Genes and Genomes
MCC	maximal clique centrality
MNC	maximum neighborhood component
OPC	oligodendrocyte progenitor cell
PCA	principal-component analysis
PPI	protein–protein interaction
PTSD	post-traumatic stress disorder
RF	random forest
ROC	receiver-operating-characteristic
SHAP	SHapley Additive exPlanations
SVM	support vector machine
