# Supplementary material for: Integrative Transcriptomics and Machine Learning Nominate IL15- and PPP2R1A-Centered Programs in PTSD Using Pathway-Informed Autonomic–Cardiac Gene Prioritization
Source: Int J Mol Sci. 2026 Sep 29;27(19):8710. doi: 10.3390/ijms27198710 (PMC13665173; doi:10.3390/ijms27198710)
Supplement: Supplementary file 1 [file ijms-27-08710-s001.zip › ijms-4542876-supplementary1_R3.pdf]

Additional file 1. Supplementary information

Integrative transcriptomics and machine learning nominate IL15- and PPP2R1A-centered programs in PTSD using pathway-informed autonomic–cardiac gene prioritization

Supplementary Methods. Original SVM selection and RF/SHAP interpretation

The original discovery workflow compared 28 eligible model or feature-selection/model combinations in the merged discovery matrix and its constituent source cohorts. SVM ranked first, with AUCs of 0.863 in the merged matrix, 0.885 in GSE63878 and 0.812 in GSE81761 (mean AUC 0.853). Because both source cohorts contributed to model development, these values represent internal consistency rather than independent external validation (Supplementary Table S3).

A separate random-forest model was used for SHAP-based biological interpretation of the 37 PPI-derived genes. The RF was tuned by 10-fold cross-validation repeated five times; mtry = 2 maximized cross-validated AUC and was used in the 500-tree interpretability model. The RF/SHAP analysis did not replace SVM as the leading original classifier (Supplementary Tables S6 and S7).

Supplementary Methods. Leakage-aware sensitivity analysis

After normalization of sample prefixes, the original files labelled train.csv and test.csv were found to contain the same underlying discovery profiles. A separate sensitivity pipeline therefore compared six classifiers using repeated nested cross-validation in the discovery matrix. Features were limited to the 29 candidate genes available in the discovery data and both external cohorts. For each candidate algorithm, hyperparameter optimization was performed within the inner cross-validation loop, and predictive performance was subsequently evaluated on the corresponding outer test folds. Algorithm performance was compared using mean outer-fold ROC AUC, after which the best-performing algorithm and its optimized hyperparameters were locked for transfer to GSE64813 and GSE97356. External outcomes were not used for feature selection, coefficient estimation, or hyperparameter refitting (Supplementary Table S5).

Supplementary Methods. External-cohort model boundary

The original external analyses fitted an rms::lrm logistic model separately in each external cohort using all candidate genes available in that cohort: 29 genes in GSE64813 and 30 in GSE97356. Predicted probabilities, ROC curves, calibration and DCA were calculated from the same samples and are therefore resubstitution/apparent performance estimates. The original plotting code used the lower and middle values of the three-value pROC confidence-interval object in the Figure 2 legend; Supplementary Table S9 reports two-sided bootstrap 2.5th and 97.5th percentiles recalculated from the archived predictions.

Supplementary Methods. Reduced nested feature-selection sensitivity analysis

To assess potential optimism arising from outcome-informed feature selection before resampling, we performed an additional reduced fully nested sensitivity analysis focused on the SVM classifier. The discovery datasets were restricted to genes shared across datasets, and no outcome-informed gene filtering was performed before outer resampling. Repeated stratified 5-fold outer cross-validation with five repetitions was used for performance estimation. Within each outer-training fold, differential-expression analysis was repeated using only the outer-training samples, with cohort included as a covariate. Candidate genes were defined using the original exploratory criterion of nominal $P < 0.05$ and $|\log2FC| > 0.1$ and were subsequently intersected with the predefined pathway-derived autonomic–cardiac gene set.

Within each outer-training fold, SVM hyperparameters were optimized using 5-fold inner cross-validation. Scaling parameters were estimated exclusively from the corresponding training partitions, and Gaussian noise augmentation (mean = 0, SD = 0.01) was restricted to training data. The optimized SVM was then refitted using the complete outer-training partition and evaluated on the untouched outer-validation fold. Predictions from the outer-validation folds were aggregated to obtain nested cross-validated estimates of discrimination (Supplementary Table S11).

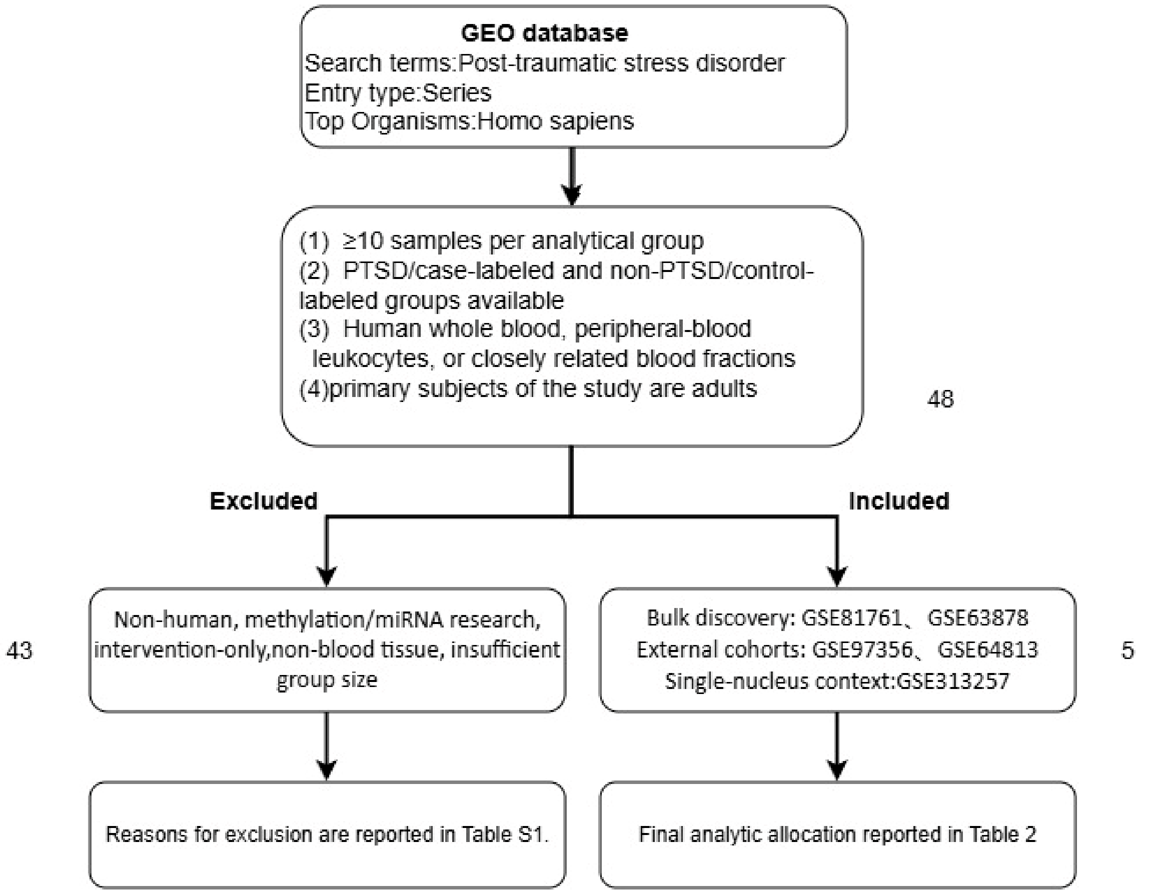

Figure S1 Flowchart of the GEO database data screening process

Table S1. GEO dataset selection

	Accession	Platform	Status	Role or reason
1	GSE276483	GPL34281	Excluded	The disease under study is traumatic brain injury,Irrelevant to the research content
2	GSE287261	GPL33022	Excluded	Methylation profiling and ketamine-intervention follow-up,Irrelevant to the research content
**3**	**GSE313257**	**GPL34284**	**Included**	**Single-nucleus context**
4	GSE288174	GPL34284	Excluded	ketamine-intervention follow-up,Irrelevant to the research content
5	GSE189587	GPL24676	Excluded	cell-model study, Sample source is not human peripheral-blood
6	**GSE63878**	**GPL6244**	**Included**	**Bulk discovery**
7	GSE216270	GPL24676	Excluded	Single-cell epigenetics research,Irrelevant to the research content
8	GSE235818	GPL18573	Excluded	Profiling Neuronal Methylome and Hydroxymethylome of Opioid Use Disorder in

				the Human Orbitofrontal Cortex, Irrelevant to the research content
9	GSE221384	GPL24676	Excluded	Compendium of functional regulatory variants in human neuropsychiatric disease,Irrelevant to the research content
10	GSE182094	GPL20301	Excluded	Compendium of functional regulatory variants in human neuropsychiatric disease, Irrelevant to the research content
11	GSE182093	GPL20301	Excluded	Compendium of functional regulatory variants in human neuropsychiatric disease, Irrelevant to the research content
12	GSE182092	GPL24676	Excluded	Compendium of functional regulatory variants in human neuropsychiatric disease,Irrelevant to the research content
13	GSE219208	GPL21697	Excluded	Sample source is hippocampal tissues not peripheral blood.
14	GSE217320	GPL16791	Excluded	iPSC-derived neurons and PBMC glucocorticoid-perturbation design, Irrelevant to the research content
15	GSE178128	GPL24676	Excluded	Methylation profiling, Irrelevant to the research content
16	GSE164877	GPL24676	Excluded	Lack of a healthy control group
17	GSE172464	GPL21145	Excluded	Methylation profiling, Irrelevant to the research content
18	GSE137624	GPL11154	Excluded	Circulating serum microRNA profiling; not mRNA expression.
19	GSE127711	GPL570	Excluded	Blood biomarkers for memory/Alzheimer disease risk, Irrelevant to the research content
20	GSE89866	GPL16791	Excluded	Blast-exposure study without PTSD case-control grouping
**21**	**GSE81761**	**GPL570**	**Included**	**Bulk discovery**
22	GSE89218	GPL13534	Excluded	DNA methylation profiling, Irrelevant to the research content
23	GSE125216	GPL570	Excluded	Stress-disorder biomarker study, Irrelevant to the research content
24	GSE127208	GPL570	Excluded	Pain-biomarker study, Irrelevant to the research content
25	GSE109409	GPL16791	Excluded	Preliminary post-deployment post-traumatic-symptom study, not suitable for research content
26	GSE114407	GPL16791	Excluded	Lack of healthy controls
27	GSE93673	GPL16791	Excluded	DNA methylation profiling, Irrelevant to the research content
28	GSE114852	GPL10558	Excluded	Cord-blood/maternal distress study, Irrelevant to the research content
**29**	**GSE97356**	**GPL11154**	**Included**	**External cohorts**
30	GSE99755	GPL16304	Excluded	Methylation/genetic-epigenetic police-officer study, Irrelevant to the research content
31	GSE76401	GPL19930	Excluded	DNA methylation profiling, Irrelevant to the

				research content
32	GSE85399	GPL10878	Excluded	DNA methylation profiling, Irrelevant to the research content
33	GSE87768	GPL17301	Excluded	Circulating microRNA profiling, Irrelevant to the research content
34	GSE83601	GPL11154	Excluded	Fewer than 10 samples
35	GSE77164	GPL10904	Excluded	The subjects of the study are children, not adults.
36	GSE74162	GPL15433	Excluded	miRNA profiling in PTSD with comorbid depression
37	GSE67663	GPL6884	Excluded	The case involves a patient with PTSD comorbid with depression, rather than a patient with PTSD alone.
38	GSE72680	GPL13534	Excluded	DNA methylation profiling,Irrelevant to the research content
39	GSE64814	GPL11154<br>GPL6244	Excluded	Irrelevant to the research content
**40**	**GSE64813**	**GPL11154**	**Included**	**External cohorts**
41	GSE199841	GPL21185	Excluded	Limited sample size relative to the dimensionality of the planned multivariable models (32 PTSD cases and 16 controls).
42	GSE52875	GPL13912<br>GPL11434<br>GPL11432	Excluded	Sample source is non-human, Irrelevant to the research content
43	GSE52872	GPL11434	Excluded	Sample source is non-human, Irrelevant to the research content
44	GSE52869	GPL11432	Excluded	Sample source is non-human, Irrelevant to the research content
45	GSE44456	GPL6244	Excluded	Stress-response pathways are altered in the hippocampus of chronic alcoholics, Irrelevant to the research content
46	GSE21282	GPL8490	Excluded	methylation profiling, Irrelevant to the research content
47	GSE860	GPL91	Excluded	not used in the harmonized modern multi-cohort workflow
48	GSE845	GPL91	Excluded	not used in the harmonized modern multi-cohort workflow

Table S2. Additional cohort clinical information

Characteristic	GSE81761	GSE63878	GSE64813	GSE97356
Age	PTSD:31.1±8.2 , controls :35.9± 7.6	PTSD:22.6±3.8, controls :23.2 ± 3.1	PTSD:22.15± 2.53 , controls: 22.42 ± 3.92	PTSD: 52.94±7.96; controls 51.36 ± 8.26
Sex	PTSD: 37 M/2 F; control: 26 M/1 F	ALL M	ALL M	ALL M
Type of trauma	military-related	military-related	military-related	World Trade Center

Characteristic	GSE81761	GSE63878	GSE64813	GSE97356
	trauma	trauma	trauma	disaster-related trauma
PTSD diagnostic criteria	NR	DSM-IV	DSM-IV	DSM-IV
timing of blood sampling	baseline	approximately 1 month before deployment and 3 months after return from deployment.	approximately 1 month before deployment and 3 months after return from deployment.	NR
treatment status	NR	NR	NR	NR
psychotropic medication use	NR	NR	NR	NR
major comorbidities	Per-sample major comorbidity fields are NR. Source-level description notes mild traumatic brain injury(TBI), depression, and sleep disorders in the cohort.	Per-sample comorbidity variables are NR in GEO	TBI: PTSD : 52/94 ; controls :42/94; major psychiatric comorbidity were not reported in the sample metadata.	Per-sample comorbidity variables are NR in GEO

NR, not reported in the original publication and/or publicly available GEO metadata

Table S3. Original machine-learning model performance across the discovery matrix and source cohorts

Model	Discovery matrix AUC	GSE63878 AUC	GSE81761 AUC	Mean AUC	SD AUC	Rank
SVM	0.8625	0.8854	0.8120	0.8533	0.0376	1
glmBoost+SVM	0.7938	0.8646	0.7051	0.7878	0.0799	2
Lasso+Stepglm[both]	0.7710	0.7969	0.7559	0.7746	0.0207	3
Enet[alpha=0.4]	0.7649	0.8025	0.7493	0.7722	0.0274	4
glmBoost+Enet[alpha=0.7]	0.7686	0.7908	0.7569	0.7721	0.0172	5

Model	Discovery matrix AUC	GSE63878 AUC	GSE81761 AUC	Mean AUC	SD AUC	Rank
Enet[alpha=0.9]	0.7660	0.7990	0.7512	0.7721	0.0245	6
Enet[alpha=0.6]	0.7646	0.8012	0.7493	0.7717	0.0267	7
glmBoost+Enet[alpha=0.2]	0.7644	0.7956	0.7550	0.7717	0.0212	8
glmBoost+Lasso	0.7686	0.7904	0.7559	0.7716	0.0174	9
Lasso+glmBoost	0.7652	0.7878	0.7616	0.7715	0.0142	10
glmBoost+Enet[alpha=0.8]	0.7678	0.7908	0.7559	0.7715	0.0177	11
glmBoost+Enet[alpha=0.4]	0.7677	0.7899	0.7569	0.7715	0.0169	12
glmBoost+Enet[alpha=0.6]	0.7667	0.7908	0.7569	0.7715	0.0174	13
glmBoost+Enet[alpha=0.1]	0.7663	0.7943	0.7531	0.7712	0.0210	14
Lasso	0.7640	0.7973	0.7521	0.7711	0.0234	15
Enet[alpha=0.5]	0.7649	0.7990	0.7493	0.7711	0.0254	16
Enet[alpha=0.3]	0.7640	0.8008	0.7483	0.7710	0.0269	17
glmBoost+Enet[alpha=0.3]	0.7658	0.7943	0.7521	0.7707	0.0215	18
glmBoost+Enet[alpha=0.9]	0.7675	0.7899	0.7540	0.7705	0.0181	19
glmBoost+Ridge	0.7629	0.7921	0.7550	0.7700	0.0195	20
glmBoost+Enet[alpha=0.5]	0.7658	0.7878	0.7550	0.7695	0.0167	21
Enet[alpha=0.8]	0.7620	0.7995	0.7417	0.7677	0.0293	22
Enet[alpha=0.2]	0.7620	0.7977	0.7426	0.7675	0.0280	23
glmBoost	0.7608	0.8008	0.7407	0.7674	0.0306	24
Enet[alpha=0.1]	0.7603	0.7921	0.7398	0.7641	0.0264	25
Lasso+SVM	0.7671	0.8229	0.6994	0.7632	0.0618	26
Enet[alpha=0.7]	0.7551	0.7925	0.7331	0.7603	0.0300	27
Ridge	0.7419	0.7600	0.7464	0.7494	0.0094	28

GSE63878 and GSE81761 contributed to construction of the merged discovery matrix; their AUCs are source-cohort internal-consistency estimates, not independent external-validation estimates.

Table S4. Venn overall list of PTSD-associated candidate genes and pathway-derived autonomic-cardiac gene set

MYL9, AIM2, MAP2K2, FASLG, STIM2, IL15, FGF17, ROCK2, KCNQ1, PIK3R3, TLR3, NCEH1, TBK1, GDF2, HTR6, CCL25, ARAP3, PPP2R5A, PSTPIP1, HGF, P2RX1, ITPKA, C5, FXYD1, ORAI1, PANX1, SHARPIN, HTR1B, GNG8, VAV3, PPP2R1A, PPP2R2B, TNNC1, GNAI2, TPCN1, PRKCA, ARHGEF1, CALML5, IL17D, GLI1, CREM, GNB2, MAP3K7, PDGFC, NPR1, ADAMTS13, OAS2, ACVR2A, BMPR1A, F8, CD70, INHA, TNFSF10, CLCF1, CHRNB2, IL11, SSTR5, JAK2, CFD, OAS1, TNFSF14, CXCR5, BCL2A1, LY96, CASP1, CALML4, CXCL8, PLA2G4A, PRKCI, PDGFD, TNFRSF12A, CSF3, CHUK, ACVR1, CXCL10, ASPH, CACNA2D3, IL34, CXCL6, CAMK1D, OAS3, CCR7, CTF1, RNASEL, GPSM3, CACNG6, PLD2, ATP1B1, IFNG, CD38, ACOX1, CASP3

Table S5. Repeated nested cross-validation model comparison

Model	Mean_AUC	SD_AUC	Median_AUC	Min_AUC	Max_AUC	N_outer_folds
Logistic_L2	0.6897	0.1174	0.6930	0.4603	0.9206	50
Linear_SVM	0.6857	0.1153	0.6905	0.4531	0.9365	50
Random_forest	0.6508	0.1301	0.6545	0.3651	0.8906	50
RBF_SVM	0.6408	0.1521	0.6369	0.2083	0.8730	50
Elastic_net	0.6313	0.1266	0.6406	0.3651	0.8889	50
Gradient_boosting	0.6308	0.1354	0.6250	0.3438	0.9048	50

Table S6. Auxiliary random-forest tuning results for SHAP analysis

mtry	Mean CV AUC	Sensitivity	Specificity	SD AUC	Selected
2	0.6785	0.6367	0.5354	0.1073	Yes
4	0.6671	0.6478	0.5543	0.1152	No
6	0.6620	0.6228	0.5721	0.1240	No
8	0.6651	0.6203	0.5675	0.1243	No
10	0.6614	0.6253	0.5557	0.1237	No

The RF was tuned using 10-fold cross-validation repeated five times. mtry = 2 was selected and used in the 500-tree RF/SHAP interpretability model. SVM remained the leading classifier in the original model ranking.

Table S7. Auxiliary random-forest SHAP results

Gene	Mean absolute SHAP	Cumulative importance	95% CI lower	95% CI upper
FASLG	0.022069	0.043346	0.019372	0.024906
IL15	0.021780	0.086123	0.019475	0.023847
OAS3	0.019142	0.123718	0.016723	0.021757
AIM2	0.018655	0.160358	0.017333	0.021226
PPP2R2B	0.017820	0.195359	0.014902	0.020707
CXCL6	0.017382	0.229498	0.015002	0.019200
CXCL8	0.017357	0.263589	0.015078	0.019531
TLR3	0.017322	0.297610	0.015142	0.018982
CCL25	0.016681	0.330373	0.015051	0.018388
FGF17	0.015452	0.360723	0.014095	0.016902

Gene	Mean absolute SHAP	Cumulative importance	95% CI lower	95% CI upper
CCR7	0.014479	0.389161	0.012305	0.016325
BCL2A1	0.014333	0.417312	0.013085	0.016073
TBK1	0.014151	0.445106	0.012972	0.015371
HGF	0.014113	0.472825	0.012853	0.015372
OAS2	0.014063	0.500445	0.012541	0.015741
CXCL10	0.013869	0.527684	0.012250	0.015654
JAK2	0.013371	0.553946	0.012285	0.014967
GPSM3	0.012896	0.579274	0.011569	0.014134
IFNG	0.012805	0.604425	0.011465	0.014427
CXCR5	0.012705	0.629379	0.011109	0.014614
CACNA2D3	0.012350	0.653636	0.010924	0.013839
CACNG6	0.012343	0.677878	0.010904	0.013902
PANX1	0.012243	0.701924	0.011402	0.013324
CD70	0.012120	0.725729	0.010708	0.013470
CD38	0.011280	0.747883	0.010294	0.012465
OAS1	0.011233	0.769946	0.009966	0.012790
LY96	0.011207	0.791958	0.010476	0.012472
PPP2R1A	0.011184	0.813924	0.010309	0.012228
RNASEL	0.010992	0.835513	0.009876	0.012050
IL11	0.010975	0.857070	0.009924	0.011746
MAP3K7	0.010925	0.878527	0.010021	0.011739
CASP3	0.010864	0.899866	0.009683	0.012020
CASP1	0.010700	0.920881	0.009876	0.011626
SHARPIN	0.010444	0.941394	0.009534	0.011324

Gene	Mean absolute SHAP	Cumulative importance	95% CI lower	95% CI upper
TNFSF10	0.010356	0.961734	0.009459	0.011418
CSF3	0.009927	0.981233	0.008815	0.010885
CHUK	0.009555	1.000000	0.008217	0.011259

Note for Tables S8–S10: For GSE64813, repeated measurements were analyzed as individual expression profiles; therefore, P values and confidence intervals should be interpreted as exploratory profile-level estimates because within-participant dependence was not modeled.

Table S8. Targeted IL15 and PPP2R1A results

Dataset	Gene	P value	AUC	AUC lower	AUC upper	Control mean	Case mean
GSE64813	IL15	0.044005	0.565584	0.478610	0.651768	7.164685	7.260330
GSE64813	PPP2R1A	0.004815	0.611985	0.528003	0.689396	10.897508	10.841708
GSE97356	IL15	0.071277	0.557828	0.479296	0.635344	8.200834	8.324716
GSE97356	PPP2R1A	0.058063	0.592531	0.517226	0.663596	8.848673	8.801204

Table S9. Original cohort-specific refitted multigene performance

Dataset	N	Cases	Controls	Candidate_genes_in_model	Apparent_AUC	Bootstrap_CI_lower	Bootstrap_CI_upper	Apparent_Brier	Fitting_boundary
GSE64813	18	94	94	29	0.8339	0.7741	0.8874	0.1663	Model refitted

Dataset	N	Cases	Controls	Candidate_genes_in_model	Apparent_AUC	Bootstrap_CI_lower	Bootstrap_CI_upper	Apparent_Brier	Fitting_boundary
	8								and evaluated in the same cohort
GSE97356	282	81	201	30	0.7487	0.6825	0.8084	0.1699	Model refitted and evaluated in the same cohort

Table S10. Locked-transfer sensitivity performance

Dataset	N	Cases	Controls	AUC	AUC_CI_lower	AUC_CI_upper	Brier	Accuracy_0.5	Sensitivity_0.5	Specificity_0.5	Calibration_intercept	Calibration_slope
Discovery_apparent	162	87	75	0.7439	0.6621	0.8167	0.2094	0.6296	0.6437	0.6133	0.1052	1.7102
GSE97356_locked_exte	28	81	201	0.53	0.4602	0.6053	0.26	0.5284	0.5556	0.5174	-0.9185	0.1842

**Dataset**	**N**	**Cases**	**Controls**	**AUC**	**AUC_CI_lower**	**AUC_CI_upper**	**Brier**	**Accuracy_0.5**	**Sensitivity_0.5**	**Specificity_0.5**	**Calibration_intercept**	**Calibration_slope**
rnal	2			41			18					
GSE64813_locked_external	188	94	94	0.6641	0.5855	0.7441	0.2309	0.6330	0.6489	0.6170	-0.0446	1.4855

Table S11. Performance and feature-selection stability in the fully nested SVM sensitivity analysis

In the reduced fully nested sensitivity analysis, all 25 outer-validation folds were successfully evaluated. The mean repeated outer-cross-validated AUC was 0.593 (SD = 0.033; range, 0.541–0.625), compared with a mean outer-fold AUC of 0.690 in the previous repeated nested evaluation conditional on the preselected 29-gene feature set. Averaging repeated out-of-fold predictions for each sample yielded a descriptive pooled AUC of 0.611 (95% CI, 0.523–0.695). Across outer folds, a median of 2,274 genes (range, 930–3,963) met the fold-specific differential-expression criterion, of which a median of 124 (range, 64–217) overlapped the predefined pathway gene set. IL15 was retained in all 25 outer folds, whereas PPP2R1A was retained in 24 of 25 folds (96%). Thus, although discrimination was attenuated under the more stringent nested validation framework, both prioritized candidates showed high selection stability across resampling iterations.

feature-selection metric	Nested sensitivity analysis result
Successful outer folds	25/25
Mean repeated nested AUC	0.593
SD	0.033
Range across 5 repeats	0.541–0.625
Pooled mean-OOF AUC	0.611
Bootstrap 95% CI	0.523–0.695
Median candidate genes/outer fold	2,274
Candidate-gene range	930–3,963
Median pathway-overlapping genes	124
Range	64–217
IL15 retention	25/25 folds (100%)
PPP2R1A retention	24/25 folds (96%)
